# Bayesian hierarchical modelling for inferring genetic interactions in yeast

**DOI:** 10.1111/rssc.12126

**Published:** 2015-10-29

**Authors:** Jonathan Heydari, Conor Lawless, David A. Lydall, Darren J. Wilkinson

**Affiliations:** ^1^ Newcastle UniversityNewcastle‐upon‐TyneUK

**Keywords:** Epistasis, Fitness, Genetic interaction, Genomics, Hierarchical models

## Abstract

Quantitative fitness analysis (QFA) is a high throughput experimental and computational methodology for measuring the growth of microbial populations. QFA screens can be used to compare the health of cell populations with and without a mutation in a query gene to infer genetic interaction strengths genomewide, examining thousands of separate genotypes. We introduce Bayesian hierarchical models of population growth rates and genetic interactions that better reflect QFA experimental design than current approaches. Our new approach models population dynamics and genetic interaction simultaneously, thereby avoiding passing information between models via a univariate fitness summary. Matching experimental structure more closely, Bayesian hierarchical approaches use data more efficiently and find new evidence for genes which interact with yeast telomeres within a published data set.

## 1 Introduction

There are many reasons to study the growth of microbes, including to prevent the growth of pathogenic bacteria or fungi and to encourage the growth of yeasts in industrial applications or during food production. Another reason is the study of eukaryotic microbes, such as the yeasts *Saccharomyces cerevisiae* (*S. cerevisiae*) and *Schizosaccharomyces pombe*, as biological models of cells in higher eukaryotes (e.g. of human cells).

Evolutionary fitness in a given environment: the probability of genetic material from an individual contributing to the gene pool of the next generation is an important characteristic of a population that is optimized by natural selection. The rate of cell division is a major component of fitness, directly affecting the ability of individuals to compete for resources such as space and nutrients. By measuring and comparing the growth rates of microbial populations (cultures) we can assess and rank the fitness or health of such populations in a given environment or in a given genetic background.

Quantitative fitness analysis (QFA) is a method for measuring the growth and fitness of independent microbial cultures inoculated onto solid agar surfaces (Banks *et al*., 2012; Addinall *et al*., 2011). During QFA we inoculate cell cultures at densities of between 96 and 1536 cultures per plate of agar, repeatedly photographing cultures as they grow, converting photographs to quantitative estimates of cell density (Lawless *et al*., 2010). We summarize observations of increasing cell density with time (growth curves) by fitting population growth models to observed data. We use fitted model parameters, such as the intrinsic growth rate parameter of the logistic growth model, to define several measures of culture fitness (Addinall *et al*., 2011).

Quantifying the fitness of hundreds of strains on a single plate, under identical environmental conditions, allows a range of powerful experimental designs. Biological experiments examining the effect of a condition on selected populations, or the effect of selected conditions on one population, are often called screens. Screening independent replicate cultures with the same genotype allows us to measure biological heterogeneity and to capture technical error (which represents the effect of measurement error, fungal and bacterial contamination, positioning errors and agar cracking in these experiments). Comparing cultures with different genotypes allows us to explore the relative importance of genes and gene products in a given environment or genetic background. An important reason for carrying out QFA is to compare the fitnesses of cultures with distinct genotypes to quantify the strength of interaction between genes (epistasis). Screening fitnesses and genetic interaction strengths on a genomewide scale allows us to study the behaviour of gene products in living cells systematically. Ready‐made genomewide libraries of strains with distinct genotypes (each with an individual gene deleted, for example) are available and can be mated with selected strains to generate libraries targeted at particular biological processes of interest. A typical high throughput, genomewide QFA screen, examining the fitness of replicate cultures of 5000 different genotypes, includes hundreds of plates that are inoculated, photographed and incubated by laboratory robots.

The genomewide QFA experiments that we reanalyse in this paper (see Section 4) were designed to inform us about telomere biology in eukaryotic cells. Telomeres are the ends of linear chromosomes found in most eukaryotic organisms (Greider and Blackburn, 1985), capping chromosome ends to ensure genetic stability, and are usually required for cells to progress through the cell cycle. Functional telomere caps help to prevent cancer and, since human telomeres shorten at each round of cell division (Olovnikov, 1973), some researchers claim that telomere‐induced replicative senescence is an important component of human aging. QFA experiments were carried out by using *S. cerevisiae* (brewer's yeast), which is a model eukaryotic organism that is widely used to study genetics. Yeasts are ideal for genomewide analysis of gene function, as genetic modification of yeast cells is relatively straightforward and yeast cultures grow quickly; millions of yeast cells can be grown overnight, whereas the same number of human cells could take weeks to grow.

In these experiments, we used a genomewide collection of *S. cerevisiae* strains, each carrying one of the set of about 5000 single open reading frame deletions that are not essential for cell survival. An open reading frame is a deoxyribonucleic acid (DNA) sequence containing no stop codons, which means that it has the potential to be translated into a protein or peptide. We refer to the mutations in this collection as *orf*Δs; Δ is the standard genetics nomenclature for a deletion. Identifying open reading frame deletions from sequences is the first step in identifying genes, and using a library of open reading frames allows the possibility of discovering biological function for sequences that were previously thought to be untranslated. However, the majority of open reading frames in the collection that we analyse have been confirmed as genes of known function and so *orf*Δs are largely equivalent to gene deletions.

The strain collection was mated with a (query) background strain carrying the *cdc13‐1* mutation, which was chosen for its relevance to telomere biology, to give a new library of strains carrying two mutations. Comparing fitnesses with a second new library of strains, built from the deletion collection mated with a strain carrying a neutral control background mutation (*ura3*Δ) allows the separation of the effect of the *cdc13‐1* mutation from that of deletions from the original collection.

More generally, we use QFA to infer genetic interaction strengths by comparing fitnesses in two QFA screens: a control screen and a query screen. All strains within a query screen differ from their control screen counterparts by a common condition such as a background gene mutation, drug treatment, temperature or other treatment. To identify strains that interact with the query condition we can compare the corresponding fitness responses for each strain in the library under the query and control conditions. Interactions with the query condition are identified by finding gene disruptions in the query screen whose fitnesses deviate significantly from those predicted by a theoretical model of genetic independence, given the fitness of corresponding gene disruptions in the control screen. Independent replicate cultures are inoculated and grown across several agar plates for each strain under each condition to capture biological heterogeneity and measurement error.

In the original analysis that was presented by Addinall *et al*. (2011), logistic models of population growth were fitted to observed cell density time courses by least squares, thereby generating a univariate fitness estimate for each time course. A linear model, predicting query strain fitness given control strain fitness, consistent with Fisher's multiplicative model of genetic independence, was used to test for genetic interaction between the query mutation and each deletion from the deletion collection. The significance of observed interactions was assigned by using a simple frequentist linear modelling approach. A major limitation of the statistical model that was used in Addinall *et al*. (2011) is that it assumes that replicate culture fitness variances are the same for each *orf*Δ. We expect that explicit modelling of heterogeneity will allow more robust identification of interactions, particularly where variability for a particular strain is unusually high (e.g. due to experimental difficulties).

Other large‐scale quantitative genetic interaction screening approaches exist, such as epistatic miniarray profiles (Schuldiner *et al*., 2006) and synthetic genetic array analysis (Tong and Boone, 2006), but we expect QFA to provide higher quality fitness estimates by using a culture inoculation technique which results in a wider range of cell densities during culture growth and by capturing complete growth curves instead of using single‐time‐point assays (Lawless *et al*., 2010). QFA as presented by Addinall *et al*. (2011) and alternative genetic interaction screening approaches mentioned above use frequentist statistical methods that cannot account for all sources of experimental variation and do not partition variation into population, genotype and repeat levels. Further, the frequentist statistical approaches that are used in the methods above cannot incorporate prior beliefs.

With the Bayesian approach (Bernardo and Smith, 2007) that we adopt in this paper, we have more flexibility of model choice, allowing us to match model structure more closely to experimental design. Bayesian analysis allows us to use binary indicators to describe the evidence that each *orf*Δ interacts with the query mutation in terms of probability. Currently there is no standard frequentist approach which can deal with inference for a hierarchical model that simultaneously models logistic growth parameters and the probability of genetic interaction. Using Bayesian hierarchical modelling (Zhang *et al*., 2014; Gelman and Hill, 2006) we look to extract as much information as possible from valuable QFA data sets.

Following the approach for determining epistasis from the comparison of two QFA screens presented by Addinall *et al*. (2011), we developed a two‐stage approach to this problem:

- a hierarchical logistic growth curve model is fitted to cell density measurements to estimate fitness; then
- fitness estimates are inputted to a hierarchical interaction model.

Next, we developed a unified approach which we refer to as the joint hierarchical model (JHM). The JHM models mutant strain fitnesses and genetic interactions simultaneously, without having to pass information between two separate models. The JHM can also allow two important, distinct, microbial fitness phenotypes (the population growth rate and carrying capacity) to provide evidence for genetic interaction simultaneously.

The paper is organized as follows: Section 2 describes the data from a typical QFA experiment. The two new models for Bayesian QFA are outlined in Section 3. In Section 4 the new Bayesian models are applied to a previously analysed QFA data set for identifying yeast genes interacting with a telomere defect. Section 5 discusses the relative merits of the newly developed Bayesian methods.

## 2 Defining fitness

**Figure 1:**
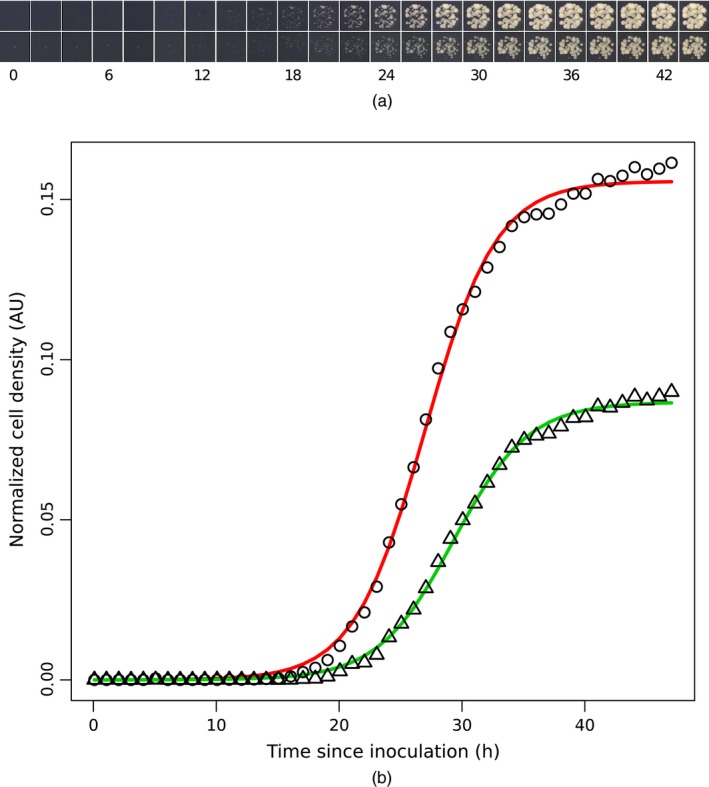
QFA image data and growth curves: (a) time lapse images for two genetically modified *S. cerevisiae* with genotypes *his*3Δ (∘, upper photographs) and *htz*1Δ (▵, lower photographs) corresponding to the time series measurements plotted in (b) time course cell density estimates derived from analysis of the time lapse images in (a) together with (least squares) fitted logistic growth curves

Observing changes in cell number in a microbial culture is the most direct way to estimate the culture growth rate, which is an important component of microbial culture fitness. Direct counting of cells in a high throughput experiment is not practical and so, during QFA, cell density estimates are made instead from culture photographs. Robotic assistance is required for both culture inoculation and image capture during genomewide screens which can include approximately 5000 independent genotypes. We use estimates of the integrated optical density generated by the image analysis tool Colonyzer (Lawless *et al*., 2010) to capture cell density dynamics in independent cultures during QFA (Fig. 1(a)).

Density estimates, scaled to normalize for camera resolution, are gathered for each culture and a dynamic model of population growth, the logistic model $\dot{x}=rx\left(1-x/K\right)$ (Verhulst, 1845), is fitted to the data. The logistic model ordinary differential equation has three parameters: *K*,* P* and *r*, the carrying capacity (maximum achievable population density), culture inoculum density (initial condition) and culture growth rate respectively, and has the following analytical solution:

(1) $$x\left(t;\theta \right)=\frac{KP\exp(rt)}{K+P\{\exp\;\left(rt\right)-1\}}P=x\left(0\right),.8pc\theta =\left(K,r,P\right).$$

This model describes self‐limiting populations undergoing approximately exponential growth which slows as the population density increases. During QFA, self‐limited growth occurs because nutrients that are found in the solid agar substrate are consumed by the growing cell population. Ultimately the population density saturates at the carrying capacity once available nutrients have been exhausted (see Fig. 1).

We can construct several distinct, quantitative fitness measures based on fitted logistic model parameters. Addinall *et al*. (2011) presented three univariate measures that are suitable for QFA: the maximum doubling rate $D_{R}$ and the maximum doubling potential $D_{P}$, and their product $D_{R}D_{P}$, where

(2) $$\begin{array}{cc} & D_{R}=\frac{r}{\log\{2\left(K-P\right)/\left(K-2P\right)\}},\\ & D_{P}=\frac{\log(K/P)}{\log(2)}. \end{array}$$

$D_{R}$ captures the rate at which microbes divide immediately after inoculation, when experiencing minimal intercellular competition or nutrient stress. A strain's growth rate largely dictates its ability to outcompete any neighbouring strains. $D_{P}$ captures the number of divisions that the culture is observed to undergo before saturation. A strain which can divide more often than its neighbours in a specific environment also has a competitive advantage.

The choice of a single overall fitness score depends on the aspects of microbial physiology that are most relevant to the biological question at hand. Typically the fitness definition $D_{R}D_{P}$ is used in QFA to account for both attributes simultaneously.

### 2.1 Epistasis

Epistasis is the phenomenon where the effects of one gene are modified by those of one or several other genes (Phillips, 1998). As presented in Addinall *et al*. (2011), here we use Fisher's multiplicative model of genetic independence (Cordell, 2002; Phenix *et al*., 2011) to represent the expected relationship between control strain fitness phenotypes and those of equivalent query strains in the absence of genetic interaction. We interpret genotypes for which the query strain fitness deviates significantly from this model of genetic independence as interacting significantly with the query mutation. Here, we use square bracket notation to represent a quantitative fitness measure. For example [*wt*] and [*query*] represent wild‐type and query mutation fitnesses respectively. *orf*Δ is standard genetics nomenclature for the genotype of a strain with a single gene *orf* deleted. We use this standard nomenclature to refer to an arbitrary strain from the deletion collection. We define new nomenclature to describe a strain containing two mutations. For example, *query:orf*Δ represents a strain with the query mutation along with an arbitrary single‐gene deletion. We use this nomenclature to refer to an arbitrary strain from the new query strain library constructed by crossing or mating a strain containing the query mutation with each of the strains in the genomewide deletion collection. Fisher's multiplicative model of genetic independence can be written as follows:

(3) [query:orfΔ]×[wt]=[query]×[orfΔ]

(4) $$\Rightarrow \left[query\;:or\;f\Delta \right]=\frac{\left[query\right]}{\left[wt\right]}\times \left[or\;f\Delta \right].$$

In expression (4), [*query*]/[*wt*] is a constant for a given pair of QFA screens, meaning that, if this model holds, there should be a linear dependence between [*query:orf*Δ] and *orf*[Δ] for all deletions *orf*Δ. During genomewide screens of thousands of independent *orf*Δs we can assume that the majority of gene mutations in the library do not interact with the chosen query mutations. Therefore, even if the query or wild‐type fitnesses are not available to us, we can still estimate the slope of this linear model by fitting it to all available fitness observations, before testing for strains which deviate significantly from the linear model. Any extra background condition, such as a gene mutation that is common to both the control and the query strains (e.g. triple‐ instead of double‐deletion strains for the query and control data sets), may change the biological interpretation of the interaction, but the same linear relationship is applicable. Besides the multiplicative model, there are other definitions for epistasis such as additive, minimum and logarithmic (Mani *et al*., 2008). Minimum is a suboptimal approach which may allow ‘masking’ of interactions (Mani *et al*., 2008). In this paper, we use a multiplicative interaction model (3), but we note that this is equivalent to an additive interaction model when looking at fitnesses on the log‐scale (Aylor and Zeng, 2008). Multiplicative and additive models are equivalent provided that fitness data are scaled appropriately (Cordell, 2002).

### 2.2 Previous quantitative fitness analysis methodology

Addinall *et al*. (2011) presented QFA where the logistic growth model (1) is fitted to experimental data by least squares to give parameter estimates $\left(\hat{K},\hat{r}\right)$ for each culture time course (each *orf*Δ replicate). The inoculum density is assumed known and the same across all *orf*Δs and their repeats. After inoculating approximately 100 cells per culture, during the first several cell divisions there are so few cells that culture cell densities remain well below the detection threshold of cameras that are used for image capture and so, without sharing information across all *orf*Δ repeats, *P* cannot be estimated directly. It is therefore necessary to fix *P* to the same value for both screens, using an average estimate of *P* from preliminary least squares logistic growth model fits. Fitting the model to each *orf*Δ repeat separately means that there is no sharing of information within an *orf*Δ or between *orf*Δs when determining $\hat{K}$ and $\hat{r}$.

Quantitative fitness scores ($F_{cm}=D_{R,\;cm}D_{P,\;cm}$) for each culture were defined (see equations (2) for definitions of $D_{R}$ and $D_{P}$). The index *c* identifies the condition for a given *orf*Δ: *c*=0 for the control strain and *c*=1 for the query strain. *m* identifies an *orf*Δ replicate. Scaled fitness measures ${\tilde{F}}_{cm}$ are calculated for both the control and the query screen such that the mean across all *orf*Δs for a given screen is equal to 1. After scaling, any evidence that ${\tilde{F}}_{0m}$ and ${\tilde{F}}_{1m}$ are significantly different will be evidence of genetic interaction.

The linear model

(5) $$\begin{array}{cc} & {\tilde{F}}_{cm}=\mu +\gamma_{c}+\varepsilon_{cm},\gamma_{0}=0,\\ & \varepsilon_{cm}\overset{\mathrm{IID}}{∼}N\left(0,\sigma^{2}\right) \end{array}$$

was fitted to the control and query strain scaled fitness measure pairs for all unique *orf*Δs in the gene deletion library. In expression (5), $\gamma_{1}$ represents the estimated strength of genetic interaction between the control and query strain. If the scaled fitnesses for the control and query strain are equivalent for a particular *orf*Δ such that they are both estimated by some *μ*, i.e. no evidence of genetic interaction, we would expect $\gamma_{c}=0$. The model was fitted by maximum likelihood, using the R function lmList (Pinheiro and Bates, 2000) with variation assumed to be the same for all strains in a given screen and the same for both control and query screens. Hence, for every gene deletion from the library an estimate of $\gamma_{1}$ was generated together with a *p*‐value for whether it was significantly different from 0.

False discovery rate corrected *q*‐values were then calculated to determine levels of significance for each *orf*Δ. Addinall *et al*. (2011) used the Benjamini–Hochberg test (Benjamini and Hochberg, 1995) for false discovery rate correction. This test is commonly used in genomic analyses as, although it assumes independence of test statistics, even if positive correlation exists between tests, the result is that false discovery rate estimates are slightly conservative. Finally a list of *orf*Δ names, ranked by *q*‐values, was outputted and *orf*Δs with *q*‐values below a significance cut‐off of 0.05 were classed as showing significant levels of genetic interaction with the query mutation.

### 2.3 Random‐effects model

We attempted to improve on the modelling approach of Addinall *et al*. (2011) within the frequentist paradigm by accounting for the hierarchical structure of the data with a random‐effects model (REM) (Pinheiro and Bates, 2000) of genetic interaction:

(6) $$\begin{array}{cc} & f_{clm}=\mu_{c}+Z_{l}+\gamma_{cl}+\varepsilon_{clm},\\ & \mu_{c}=\left\{\begin{array}{cc} \mu +\alpha & \text{if}\;c=0,\\ \mu & \text{if}\;c=1, \end{array}\right.\\ & \gamma_{cl}=\left\{\begin{array}{cc} 0 & \text{if}\;c=0,\\ \gamma_{l} & \text{if}\;c=1, \end{array}\right.\\ & Z_{l}∼N\left(0,\sigma_{Z}^{2}\right),\\ & \varepsilon_{clm}∼N\left(0,\sigma^{2}\right). \end{array}$$

In the REM (6) and in models presented below, *c* identifies the condition for a given *orf*Δ, *l* identifies a particular *orf*Δ from the gene deletion library and *m* identifies a repeat for a given *orf*Δ. In expression (6) we use previously estimated $F_{cm}$ to quantify interaction for all *orf*Δs simultaneously. Introducing a random effect $Z_{l}$ allows us to account for between‐subject variation by estimating a single $\sigma_{Z}^{2}$. Unlike the approach of Addinall *et al*. (2011), we do not scale the observed values $F_{clm}$ and instead introduce a parameter to model a condition effect $\mu_{c}$. $\gamma_{cl}$ represents the estimated strength of genetic interaction between an *orf*Δ and our query mutation. For a multiplicative model of epistasis we use an additive model to describe log‐transformed data $f_{clm}=\log\left(F_{clm}+1\right)$, where $F_{clm}$ are our observed fitnesses. We use the Benjamini–Hochberg test to correct for multiple testing to make a fair comparison with the approach of Addinall *et al*. (2011).

We find that *orf*Δ level variation in fitness cannot be modelled efficiently as random effects under the frequentist paradigm, which forces us to assume constant variance for all *orf*Δs. The large number of random effects required (control and query observations for each of about 5000 *orf*Δs in a genomewide screen) to model variances at the *orf*Δ level resulted in inference involving large matrix computations that either took too long to complete or were not possible by using the computing hardware that was available to us. Similarly we found that it is not practical to model genetic interaction and cell population growth curves simultaneously as random effects under the frequentist paradigm. We attempted to model repeat level variation with a normal distribution by fitting a model with a log‐link function; however, none of the non‐linear model maximum likelihood algorithms that we tried converged.

## 3 Bayesian hierarchical model inference

As an alternative to the maximum likelihood approach that was presented by Addinall *et al*. (2011) and the REM, we present a Bayesian hierarchical methodology where *a priori* uncertainty about each parameter value is described by probability distributions (Bernardo and Smith, 2007) and information about parameter distributions is shared across *orf*Δs and conditions. Plausible frequentist estimates from across 10 independent, unpublished QFA data sets, including a wide range of background mutations and treatments were summarized to establish and quantify our *a priori* uncertainty in model parameters.

First and foremost, prior distributions describe our beliefs about parameter values. Priors should be at least sufficiently diffuse to capture all plausible values (to capture the full range of observations in the data sets) and at least sufficiently restrictive to rule out physically implausible values (to ensure efficient inference). Priors that are excessively vague are not consistent with the Bayesian paradigm and if they are unnecessarily diffuse can also result in computational difficulties during inference (see below for further details). The computational time that is required to overcome mixing problems from a careless choice of prior distributions is likely to be considerable when fitting a large hierarchical model to a rich data set. Although using conjugate priors would allow slightly faster inference, we find that, for this particular application, the conjugate priors that are available for variance parameters (Gelman, 2006) are either too restrictive at low variance (inverse gamma), not sufficiently restrictive at low variance (half *t* family of prior distributions) or are non‐informative or largely discard the prior information that is available (uniform). Here we have chosen the non‐conjugate log‐normal distribution as a prior for precision parameters as we find that when appropriately parameterized the distribution reflects our prior beliefs about precision parameters and is only restrictive at extremely high and low variances.

We use three types of distribution to model parameter uncertainty: the log‐normal, normal and scaled *t*‐distribution with 3 degrees of freedom. Particular care is needed in the choice of distributions for parameters which are in some sense close to the data, to ensure that the model is sufficiently flexible to describe high resolution data sets such as those captured during QFA. We use the log‐normal distribution to describe parameters which are required to be non‐negative (e.g. parameters describing precisions, or repeat level fitnesses) or parameter distributions which are found by visual inspection to be asymmetric. We use the normal distribution to describe parameters which are symmetrically distributed (e.g. some prior distributions and the measurement error model) and we use the *t*‐distribution to describe parameters whose uncertainty distribution is long tailed (i.e. where using the normal distribution would result in excessive shrinkage towards the mean). For example, after visual inspection of the variation of frequentist *orf*Δ level means about their population means in historical data sets, we found many unusually fit, dead or missing *orf*Δs and concluded that *orf*Δ fitnesses would be well modelled by the *t*‐distribution.

Instead of manually fixing the inoculum density parameter *P* as in Addinall *et al*. (2011) our Bayesian hierarchical models deal with the scarcity of information about the early part of culture growth curves by estimating a single *P* across all *orf*Δs (and conditions in some of our models). Our new approach learns about *P* from the data and gives us a posterior distribution to describe our uncertainty about its value.

The new hierarchical structure (Goldstein, 2011) that was implemented in our models reflects the structure of QFA experiments. Information is shared efficiently among groups of parameters, such as between repeat level parameters for a single mutant strain. Examples of the type of Bayesian hierarchical modelling which we use to model genetic interaction can be seen in Zhang *et al*. (2014) and Yi (2010), where hierarchical models are used to account for group effects.

In Phenix *et al*. (2011) the signal of genetic interaction is chosen to be ‘strictly on or off’ when modelling gene activity. We include this concept in our interaction models by using the posterior probability of a Bernoulli‐distributed indicator variable (O'Hara and Sillanpaa, 2009) to describe whether there is evidence of an *orf*Δ interacting with the query mutation; the more evidence of interaction, the closer posterior expectations will be to 1.

Failing to account for all sources of variation within the experimental structure, such as the difference in variation between the control and query fitnesses, may lead to inaccurate conclusions. By incorporating more information into the model with prior distributions and a more flexible modelling approach, we shall increase statistical power. With an improved analysis it may then be possible for a similar number of genetic interactions to be identified with a smaller sample size (fewer replicate cultures), saving on the experimental costs that are associated with QFA.

Inference is carried out by using Markov chain Monte Carlo methods. The algorithm that was used is a Metropolis‐within‐Gibbs sampler where each full conditional is sampled in turn either directly or by using a simple normal random‐walk Metropolis step. The scheme that was used is similar to that presented by Jow *et al*. (2014). Owing to the large number of model parameters and the large quantity of data from high throughput QFA experiments, the algorithms that are used for carrying out inference often have poor mixing and give highly auto‐correlated samples, requiring thinning. Posterior means are used to obtain point estimates where required.

In what follows, we present a two‐stage Bayesian, hierarchical modelling approach (Section 3.1 and 3.2) where we generate *orf*Δ fitness distributions and infer genetic interaction probabilities separately. We then present a one‐stage approach (Section 3.3) for inferring fitness and genetic interaction probabilities simultaneously. For the new approaches that are described in Section 3.1, 3.2 and 3.3 model fitting is carried out by using the techniques discussed above, implemented in C for computational speed, and the code is freely available in the R package qfaBayes at https://rforge.rproject.org/projects/qfa.

For the Bayesian models presented, the flow of information within the models and how each parameter is related to the data can be seen from the plate diagrams in Section 1 of the on‐line supporting materials.

### 3.1 Separate hierarchical model

The separate hierarchical model (SHM), given in expression (7), models the growth of multiple yeast cultures by using the logistic model described in equation (1), whose analytic solution is indicated by *x*(*t*). The observational model at the time point level is given by

$$\begin{array}{cc} & y_{lmn}∼N\left\{{\hat{y}}_{lmn},{\left(\nu_{l}\right)}^{-1}\right\},\\ & {\hat{y}}_{lmn}=x\left(t_{lmn};K_{lm},r_{lm},P\right), \end{array}$$

where *l*=1,2,…,*L* (*orf*Δ level), $m=1,\ldots ,M_{l}$ (repeat level) and $n=1,2,\ldots ,N_{lm}$ (time point level). At the next level of the hierarchy (the repeat level), we have

$$\begin{array}{cc} & \log\left(K_{lm}\right)∼N\left\{K_{l}^{o},{\left(\tau_{l}^{K}\right)}^{-1}\right\}I_{\left(-\infty ,\;0\right]},\log\left(\tau_{l}^{K}\right)∼N\left\{\tau^{K,\;p},{\left(\sigma^{\tau ,\;K}\right)}^{-1}\right\}I_{\left[0,\;\infty \right)},\\ & \log\left(r_{lm}\right)∼N\left\{r_{l}^{o},{\left(\tau_{l}^{r}\right)}^{-1}\right\}I_{\left(-\infty ,\;3.5\right]},\;\;\log\left(\tau_{l}^{r}\right)∼N\left\{\tau^{r,\;p},{\left(\sigma^{\tau ,\;r}\right)}^{-1}\right\}. \end{array}$$

Moving up, at the *orf*Δ level we have

$$\begin{array}{cc} & \exp\;\left(K_{l}^{o}\right)∼t\left\{K^{p},{\left(\sigma^{K,\;o}\right)}^{-1},3\right\}I_{\left[0,\;\infty \right)},\log\left(\sigma^{K,\;o}\right)∼N\left\{\eta^{K,\;o},{\left(\psi^{K,\;o}\right)}^{-1}\right\},\\ & \exp\;\left(r_{l}^{o}\right)∼t\left\{r^{p},{\left(\sigma^{r,\;o}\right)}^{-1},3\right\}I_{\left[0,\;\infty \right)},\log\left(\sigma^{r,\;o}\right)∼N\left\{\eta^{r,\;o},{\left(\psi^{r,\;o}\right)}^{-1}\right\},\\ & \log\left(\nu_{l}\right)∼N\left\{\nu^{p},{\left(\sigma^{\nu }\right)}^{-1}\right\},\log\left(\sigma^{\nu }\right)∼N\left\{\eta^{\nu },{\left(\psi^{\nu }\right)}^{-1}\right\}. \end{array}$$

Finally, at the population level, we take

(7) $$\begin{array}{cc} & \log\left(K^{p}\right)∼N\left\{K^{\mu },{\left(\eta^{K,\;p}\right)}^{-1}\right\},\\ & \log\left(r^{p}\right)∼N\left\{r^{\mu },{\left(\eta^{r,\;p}\right)}^{-1}\right\},\\ & \log\left(P\right)∼N\{P^{\mu },{\left(\eta^{P}\right)}^{-1}\},\\ & \nu^{p}∼N\left\{\nu^{\mu },{\left(\eta^{\nu ,\;p}\right)}^{-1}\right\},\\ & \tau^{K,\;p}∼N\left\{\tau^{K,\;\mu },{\left(\eta^{\tau ,\;K,\;p}\right)}^{-1}\right\},\\ & \log\left(\sigma^{\tau ,\;K}\right)∼N\left\{\eta^{\tau ,\;K},{\left(\psi^{\tau ,\;K}\right)}^{-1}\right\},\\ & \tau^{r,\;p}∼N\left\{\tau^{r,\;\mu },{\left(\eta^{\tau ,\;r,\;p}\right)}^{-1}\right\},\\ & \log\left(\sigma^{\tau ,\;r}\right)∼N\left\{\eta^{\tau ,\;r},{\left(\psi^{\tau ,\;r}\right)}^{-1}\right\}. \end{array}$$

Dependent variable observations $y_{lmn}$ (scaled cell density measurements) and independent variable $t_{lmn}$ (the time since inoculation) are model inputs, where *n* indicates the time point for a given *orf*Δ repeat. A directed acyclic graph for this model can be seen in section 1 of the supporting on‐line information. In this first hierarchical model, the logistic model is fitted to query and control data separately.

To measure the variation between *orf*Δs, parameters ($K^{p}$, $\sigma_{o}^{K}$) and ($r^{p}$,$\sigma_{o}^{r}$) are included at the population level of the hierarchy. Within‐*orf*Δ variation is modelled by each set of *orf*Δ level parameters ($K_{l}^{o}$, $\tau_{l}^{K}$) and ($r_{l}^{o}$,$\tau_{l}^{r}$). Learning about these higher level parameters allows information to be shared across parameters that are lower in the hierarchy. A three‐level hierarchical model is applied to $\left(K,K_{l}^{o},K_{lm}\right)$ and $\left(r,r_{l}^{o},r_{lm}\right)$, sharing information on the repeat level and the *orf*Δ level. Note that *orf*Δ level parameters $K_{l}^{o}$ and $r_{l}^{o}$ are on the log‐scale ($\exp\;\left(K_{l}^{o}\right)$ and $\exp\;\left(r_{l}^{o}\right)$ are on the scale of the observed data).

Assuming a normal error structure, random measurement error is modelled by the $\nu_{l}$‐parameters (one for each *orf*Δ). Information on random error is shared across all *orf*Δs by drawing $\log(\nu_{l})$ from a normal distribution parameterized by ($\nu_{p}$, $\sigma^{\nu }$). A two‐level hierarchical structure is also used for both the $\tau_{l}^{K}$‐ and the $\tau_{l}^{r}$‐parameters.

Modelling logistic model parameter distributions on the log‐scale ensures that parameter values remain strictly positive (a realistic biological constraint). Truncating distributions allows us to implement further realistic constraints on the data. Truncating $\log(r_{lm})$ values greater than 3.5 corresponds to disallowing biologically unrealistic culture doubling times faster than about 30 min and truncating of repeat level parameters $\log(K_{lm})$ above 0 ensures that no carrying capacity estimate is greater than the maximum observable cell density, which is 1 after scaling.

*orf*Δ level parameters $\exp\;\left(K_{l}^{o}\right)$ and $\exp\;\left(r_{l}^{o}\right)$ are on the same scale as the observed data. Realistic biological constraints (positive logistic model parameters) are enforced at the repeat level; however, both $\exp\;\left(K_{o}^{l}\right)$ and $\exp\;\left(r_{o}^{l}\right)$, which are assumed to have scaled *t*‐distributions, are truncated below 0 to keep exponentiated parameters strictly positive.

Identifiability problems can arise for parameters $K_{lm}$ and $r_{lm}$ when observed cell densities are low and unchanging (consistent with growth curves for cultures which are very sick, dead or missing). In these cases, either $K_{lm}$ or $r_{lm}$ can take values near 0, allowing the other parameter to take any value without significantly affecting the model fit. In the approach of Addinall *et al*. (2011) identification problems are handled in an automated post‐processing stage: for cultures with low *K*‐estimates (classified as dead), *r* is automatically set to 0. Computing time wasted on such identifiability problems is reduced by truncating repeat level parameters $r_{lm}$, preventing the Markov chain Monte Carlo algorithms from becoming stuck in extremely low probability regions when $K_{lm}$ takes nearly 0 values. Similarly, $\log(\tau_{l}^{K})$ parameters are truncated below 0 to overcome identifiability problems between parameters $K_{lm}$ and $r_{lm}$ when $r_{lm}$ takes nearly 0 values.

The SHM (7) is fitted to both the query and the control strains separately. Means are taken to summarize logistic growth parameter posterior distributions. Summaries $\left({\hat{K}}_{lm},{\hat{r}}_{lm},\hat{P}\right)$ for each *orf*Δ repeat are converted to univariate fitnesses $F_{clm}$ where *c* identifies the condition (query or control), with any given fitness measure, e.g. $D_{R}D_{P}$ (see equation (2) and Addinall *et al*. (2011)).

### 3.2 Interaction hierarchical model

After the SHM fit, the interaction hierarchical model (IHM), given in expression (8), can then be used to model estimated fitness scores $F_{clm}$ and to determine, for each *orf*Δ, whether there is evidence for interaction: *c*=0,1 (condition level), $l=1,\ldots ,L_{c}$ (*orf*Δ level) and $m=1,\ldots ,M_{cl}$ (repeat level). At the repeat level,

$$\begin{array}{cc} & F_{clm}∼N\left\{{\hat{F}}_{cl},{\left(\nu_{cl}\right)}^{-1}\right\},\\ & {\hat{F}}_{cl}=\exp\left(\alpha_{c}+Z_{l}+\delta_{l}\gamma_{cl}\right); \end{array}$$

at the *orf*Δ level,

$$\begin{array}{cc} & \exp\left(Z_{l}\right)∼t\left\{Z^{p},{\left(\sigma^{Z}\right)}^{-1},3\right\}I_{\left[0,\;\infty \right)},\log\left(\sigma^{Z}\right)∼N\left(\eta^{Z},\psi^{Z}\right),\\ & \log\left(\nu_{cl}\right)∼N\left\{\nu^{p},{\left(\sigma^{\nu }\right)}^{-1}\right\},\log\left(\sigma^{\nu }\right)∼N\left(\eta^{\nu },\psi^{\nu }\right),\\ & \delta_{l}∼\text{Bern}\left(p\right),\\ & \exp\left(\gamma_{cl}\right)=\left\{\begin{array}{cc} 1 & \;\text{if}\;c=0,\\ t\left\{1,{\left(\sigma^{\gamma }\right)}^{-1},3\right\}I_{\left[0,\;\infty \right)} & \text{if}\;c=1, \end{array}\right.\\ & \log\left(\sigma^{\gamma }\right)∼N\left\{\eta^{\gamma },{\left(\psi^{\gamma }\right)}^{-1}\right\}; \end{array}$$

at the condition level,

$$\alpha_{c}=\left\{\begin{array}{cc} 0 & \text{if}\;c=0,\\ N(\alpha^{\mu },\eta^{\alpha }) & \text{if}\;c=1; \end{array}\right.$$

at the population level,

(8) $$\begin{array}{cc} & \log\left(Z^{p}\right)∼N\left\{Z^{\mu },{\left(\eta^{Z,\;p}\right)}^{-1}\right\},\\ & \nu^{p}∼N\left\{\nu^{\mu },{\left(\eta^{\nu ,\;p}\right)}^{-1}\right\}. \end{array}$$

$F_{clm}$ are the observed fitness scores. A directed acyclic graph for this model can be found in section 1 of the supporting on‐line materials. Fitnesses are passed to the IHM where query screen fitnesses are compared with control screen fitnesses, assuming genetic independence. Deviations from predicted fitnesses are evidence for genetic interaction. The interaction model accounts for between‐*orf*Δ variation with the set of parameters ($Z^{p}$,$\sigma_{Z}$) and within‐*orf*Δ variation by the set of parameters ($Z_{l}$,$\nu_{l}$). A linear relationship between the control and query *orf*Δ level parameters is specified with a scale parameter $\alpha_{1}$. Deviation from this relationship (genetic interaction) is accounted for by the term $\delta_{l}\gamma_{1l}$. A scaling parameter $\alpha_{1}$ allows any effects due to differences in the control and query data sets to be scaled out, such as differences in genetic background, incubator temperature or inoculum density. The Bernoulli probability parameter *p* is our prior estimate for the probability of a given *orf*Δ showing evidence of genetic interaction. For the data set that is considered in Section 4 *p* is set to 0.05 as the experimenter's belief before the experiment was carried out was that 5% of the *orf*Δs would interact with the query. Observational noise is quantified by $\nu_{cl}$. The $\nu_{cl}$‐parameter accounts for a difference in variation between condition, i.e. the query and control data sets, and for a difference in variation between *orf*Δs.

The linear relationship between the control and query fitness scores, consistent with the multiplicative model of genetic independence, described in expression (4), is implemented in the IHM as $\hat{F}=\exp\;\left(\alpha_{c}+Z_{l}+\delta_{l}\gamma_{cl}\right)=\exp\;\left(\alpha_{c}\right)\exp\;\left(Z_{l}+\delta_{l}\gamma_{cl}\right)$. Strains whose fitnesses lie along the linear relationship defined by the scalar $\alpha_{1}$ show no evidence for interaction with the query condition. In contrast, deviation from the linear relationship, represented by the posterior mean of $\delta_{l}\gamma_{1l}$, is evidence for genetic interaction. The larger the posterior mean for $\delta_{l}$ is, the higher the probability or evidence there is for interaction, whereas $\gamma_{1l}$ is a measure of the strength of interaction. Where the query condition has a negative effect (i.e. decreases fitness on average, compared with the control condition), query fitnesses which are above and below the linear relationship are suppressors and enhancers of the fitness defect that is associated with the query condition respectively. A list of genes ranked by strength and direction of interaction with the query condition is ordered by the posterior means of $\delta_{l}\gamma_{cl}$. The *orf*Δs with ${\hat{\delta }}_{l}>0.5$ are classified and labelled as showing ‘significant’ evidence of interaction.

### 3.3 Joint hierarchical model

The JHM, given in expression (9), is an alternative, fully Bayesian version of the two‐stage approach that was described in Sections 3.1 and 3.2: *c*=0,1 (condition level), $l=1,\ldots ,L_{c}$ (*orf*Δ level), $m=1,\ldots ,M_{cl}$ (repeat level) and $n=1,\ldots ,N_{clm}$ (time point level). At the time point level,

$$\begin{array}{cc} & y_{clmn}∼N\left\{{\hat{y}}_{clmn},{\left(\nu_{cl}\right)}^{-1}\right\},\\ & {\hat{y}}_{clmn}=x\left(t_{clmn};K_{clm},r_{clm},P\right); \end{array}$$

at the repeat level,

$$\begin{array}{cc} & \log\left(K_{clm}\right)∼N\left\{\alpha_{c}+K_{l}^{o}+\delta_{l}\gamma_{cl},{\left(\tau_{cl}^{K}\right)}^{-1}\right\}I_{\left(-\infty ,\;0\right]},\;\log\left(\tau_{cl}^{K}\right)∼N\left\{\tau_{c}^{K,\;p},{\left(\sigma_{c}^{\tau ,\;K}\right)}^{-1}\right\}I_{\left[0,\;\infty \right)},\;\;\\ & \log\left(r_{clm}\right)∼N\left\{\beta_{c}+r_{l}^{o}+\delta_{l}\omega_{cl},{\left(\tau_{cl}^{r}\right)}^{-1}\right\}I_{\left(-\infty ,\;3.5\right]},\log\left(\tau_{cl}^{r}\right)∼N\left\{\tau_{c}^{r,\;p},{\left(\sigma_{c}^{\tau ,\;r}\right)}^{-1}\right\}; \end{array}$$

at the *orf*Δ level,

$$\begin{array}{cc} & \exp\left(K_{l}^{o}\right)∼t\left\{K^{p},{\left(\sigma^{K,\;o}\right)}^{-1},3\right\}I_{\left[0,\;\infty \right)},\log\left(\sigma^{K,\;o}\right)∼N\left\{\eta^{K,\;o},{\left(\psi^{K,\;o}\right)}^{-1}\right\},\\ & \exp\left(r_{l}^{o}\right)∼t\left\{r^{p},{\left(\sigma^{r,\;o}\right)}^{-1},3\right\}I_{\left[0,\;\infty \right)},\log\left(\sigma^{r,\;o}\right)∼N\left\{\eta^{r,\;o},{\left(\psi^{r,\;o}\right)}^{-1}\right\},\\ & \log\left(\nu_{cl}\right)∼N\left\{\nu^{p},{\left(\sigma^{\nu }\right)}^{-1}\right\},\log\left(\sigma^{\nu }\right)∼N\left\{\eta^{\nu },{\left(\psi^{\nu }\right)}^{-1}\right\},\\ & \delta_{l}∼\text{Bern}\left(p\right),\\ & \exp\left(\gamma_{cl}\right)=\left\{\begin{array}{cc} 1 & \text{if}\;c=0,\\ t\left\{1,{\left(\sigma^{\gamma }\right)}^{-1},3\right\}I_{\left[0,\;\infty \right)} & \text{if}\;c=1, \end{array}\right.\\ & \log\left(\sigma^{\gamma }\right)∼N\left(\eta^{\gamma },\psi^{\gamma }\right),\\ & \exp\;\left(\omega_{cl}\right)=\left\{\begin{array}{cc} 1 & \text{if}\;c=0,\\ t\{1,\left({\sigma^{\omega })}^{-1},3\right\}I_{\left[0,\;\infty \right)} & \text{if}\;c=1, \end{array}\right.\\ & \log\left(\sigma^{\omega }\right)∼N\left(\eta^{\omega },\psi^{\omega }\right); \end{array}$$

at the condition level,

$$\begin{array}{cc} \alpha_{c} & =\left\{\begin{array}{cc} 0 & \text{if}\;c=0,\\ N(\alpha^{\mu },\eta^{\alpha }) & \;\text{if}\;c=1, \end{array}\right.\\ & \beta_{c}=\left\{\begin{array}{cc} 0 & \;\text{if}\;c=0,\\ N(\beta^{\mu },\eta^{\beta }) & \;\text{if}\;c=1, \end{array}\right.\\ & \tau_{c}^{K,\;p}∼N\left\{\tau^{K,\;\mu },{\left(\eta^{\tau ,\;K,\;p}\right)}^{-1}\right\},\\ & \log\left(\sigma_{c}^{\tau ,\;K}\right)∼N\left\{\eta^{\tau ,\;K},{\left(\psi^{\tau ,\;K}\right)}^{-1}\right\},\\ & \tau_{c}^{r,\;p}∼N\left\{\tau^{r,\;\mu },{\left(\eta^{\tau ,\;r,\;p}\right)}^{-1}\right\},\\ & \log\left(\sigma_{c}^{\tau ,\;r}\right)∼N\left\{\eta^{\tau ,\;r},{\left(\psi^{\tau ,\;r}\right)}^{-1}\right\}; \end{array}$$

at the population level,

(9) $$\begin{array}{cc} & \log\left(K^{p}\right)∼N\left\{K^{\mu },{\left(\eta^{K,\;p}\right)}^{-1}\right\},\\ & \log\left(r^{p}\right)∼N\left\{r^{\mu },{\left(\eta^{r,\;p}\right)}^{-1}\right\},\\ & \nu^{p}∼N\left\{\nu^{\mu },{\left(\eta^{\nu ,\;p}\right)}^{-1}\right\},\\ & \log\left(P\right)∼N\{P^{\mu },{\left(\eta^{P}\right)}^{-1}\}. \end{array}$$

Here, the dependent variable $y_{clmn}$ (scaled cell density measurements) and independent variable $t_{clmn}$ (the time since inoculation) are inputted to the JHM. The JHM incorporates the key modelling ideas from both the SHM and the IHM with the considerable advantage that we can learn about logistic growth model, fitness and genetic interaction parameters simultaneously, thereby avoiding having to choose a fitness measure or point estimates for passing information between models. The JHM is an extension of the SHM with the presence or absence of genetic interaction being described by a Bernoulli indicator and an additional level of error to account for variation due to the query condition. Genetic interaction is modelled in terms of the two logistic growth parameters *K* and *r* simultaneously.

By fitting a single JHM, we need only to calculate posterior means, to check model diagnostics and to thin posterior samples once. However, the computing time taken to reach convergence for any given data set is roughly twice that of the two‐stage approach for a genomewide QFA.

All of the SHM and IHM modelling assumptions that were described in Sections 3.1 and 3.22, such as distributional choices and hierarchical structure, are inherited by the JHM. Similarly to the interaction model in Section 3.2, linear relationships between control and query carrying capacity and growth rate (instead of fitness score) are assumed: $\left(\exp\;\left(\alpha_{c}+K_{l}^{o}+\delta_{l}\gamma_{cl}\right),\exp\;\left(\beta_{c}+r_{l}^{o}+\delta_{l}\omega_{cl}\right)\right)$.

## 4 Reanalysis of quantitative fitness analysis experiments designed to learn about telomere biology

In this section we present a reanalysis of a previously published experiment, designed to inform us about the ways that eukaryotic cells respond to the loss of telomere caps that normally protect the ends of chromosomes from being erroneously recognized as a type of DNA damage. A pair of genomewide QFA screens were carried out in the model eukaryotic organism *S. cerevisiae* (brewer's yeast), comparing the fitness of control *ura3*Δ strains with query *cdc13‐1* strains. These comparisons were made to identify genes that show evidence of interaction with the query mutation *cdc13‐1*. CDC13 is an *S. cerevisiae* protein which binds to telomeres and regulates telomere capping. *cdc13‐1* is a temperature‐sensitive allele of the CDC13 gene. The ability of the altered *cdc13* protein produced by strains carrying the *cdc13‐1* gene to cap telomeres is reduced at temperatures above $26{\;}^{\circ }C$ (Nugent *et al*., 1996), inducing a fitness defect that can be measured by QFA. The original experimental data that were used are freely available from http://research.ncl.ac.uk/colonyzer/AddinallQFA/. Addinall *et al*. (2011) presented a list of inferred interaction strengths and *p*‐values for significance of interaction, together with a fitness plot for this experiment.

Here, we shall compare lists of genes classified as interacting with *cdc13‐1* by the non‐hierarchical frequentist approach that was presented by Addinall *et al*. (2011) and the hierarchical REM with those classified as interacting by our hierarchical Bayesian approaches.

4294 non‐essential *orf*Δs were selected from the yeast deletion collection and used to build the corresponding double‐deletion control and query strains. Independent replicate culture growth curves (time course observations of cell density) were captured for each control and query strain. The median and range for the number of replicates per *orf*Δ are 8 and [8,144] respectively. The range for the number of time points for growth curves captured in the control experiment is [7,22] and [9,15] in the query experiment.

As in the analysis of Addinall *et al*. (2011), a list of 159 genes are stripped from our final list of genes for biological and experimental reasons. Priors for the models used throughout Section 4 are provided in Table 1. We have ensured that these priors are sufficiently diffuse to describe any QFA data set by inspecting 10 historical QFA data sets.

**Table 1:** Hyperparameter values specifying priors for the SHM, IHM and JHM

*Results for the SHM and JHM*		*Results for the SHM and JHM*		*Results for SHM and JHM*		*Results for SHM and JHM*	
*Parameter name*	*Value*	*Parameter name*	*Value*	*Parameter name*	*Value*	*Parameter name*	*Value*
$\tau^{K,\mu }$	2.20	$\eta^{r,p}$	0.13	$\alpha^{\mu }$	0.00	$Z_{\mu }$	3.66
$\eta^{\tau ,K,p}$	0.02	$\nu^{\mu }$	19.82	$\eta^{\alpha }$	0.25	$\eta^{Z,p}$	0.70
$\eta^{K,o}$	−0.79	$\eta^{\nu ,p}$	0.02	$\beta^{\mu }$	0.00	$\eta^{Z}$	0.10
$\psi^{K,o}$	0.61	$P^{\mu }$	−9.04	$\eta^{\beta }$	0.25	$\psi^{Z}$	0.42
$\tau^{r,\mu }$	3.65	$\eta^{P}$	0.47	*p*	0.05	$\eta^{\nu }$	0.10
$\eta^{\tau ,r,p}$	0.02			$\eta^{\gamma }$	−0.79	$\psi^{\nu }$	2.45
$\eta^{r,o}$	0.47			$\psi^{\gamma }$	0.61	$\nu^{\mu }$	2.60
$\psi^{r,o}$	0.10			$\eta^{\omega }$	0.47	$\eta^{\nu ,p}$	0.05
$\eta^{\nu }$	−0.83			$\psi^{\omega }$	0.10	$\alpha^{\mu }$	0.00
$\psi^{\nu }$	0.86			$\eta^{\tau ,K}$	2.20	$\eta^{\alpha }$	0.31
$K^{\mu }$	−2.01			$\psi^{\tau ,K}$	0.02	*p*	0.05
$\eta^{K,p}$	0.03			$\eta^{\tau ,r}$	3.65	$\eta^{\gamma }$	0.10
$r^{\mu }$	0.97			$\psi^{\tau ,r}$	0.02	$\psi^{\gamma }$	0.42

### 4.1 Model application

The Heidelberger–Welch (Heidelberger and Welch, 1981) and Raftery–Lewis (Raftery and Lewis, 1995) convergence diagnostics are used to determine whether convergence has been reached for all parameters. Posterior and prior densities are compared by eye to ensure that sample posterior distributions are not restricted by the choice of prior distribution. Auto‐correlation function plot diagnostics are checked visually to ensure that serial correlation between sample values of the posterior distribution is low, ensuring that the effective sample size is similar to the actual sample size.

**Figure 2:**
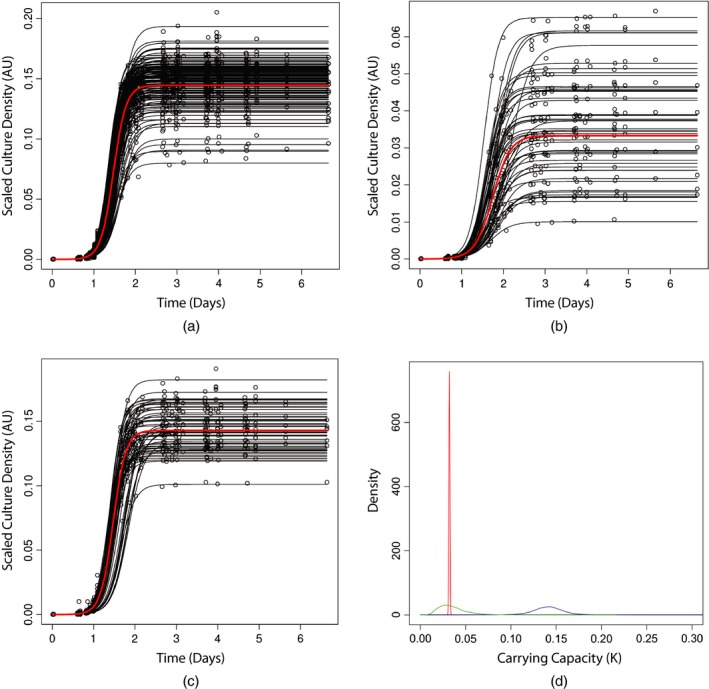
Hierarchy of model fits and parameter estimates (data for *orf*Δ repeats have been plotted in (a), (b) and (c) with SHM‐fitted curves overlaid in black for repeat level parameters and red for the *orf*Δ level parameter fit): (a) SHM scatter plot for 144 *his3*Δ *ura3*Δ repeats at $27^{\;\circ }$C; (b) SHM scatter plot for 48 *rad50*Δ *ura3*Δ repeats at 27${}^{\;\circ }$C; (c) SHM scatter plot for 56 *exo1*Δ *ura3*Δ repeats at $27^{\;\circ }$C; (d) SHM density plot of posterior predictive distributions for *rad50*Δ *ura3*Δ carrying capacity *K* hierarchy (the prior distribution for $K^{p}$ is flat over this range; parameters $K^{p}$, $\text{exp}(K_{l}^{o})$ and $K_{clm}$ are on the same scale as the observed data) (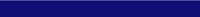, posterior predictive for $\text{exp}(K_{l}^{o})$; 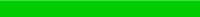, posterior predictive for $K_{clm}$; 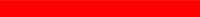, posterior distribution of the first time course repeat $K_{clm}$‐parameter)

To assess how well the logistic growth model describes cell density observations we generate plots of raw data with fitted curves overlaid. Figs 2(a), 2(b) and 2(c) show time series data for three different mutant strain repeats at $27\;\;{}^{\circ }$C, together with fitted logistic curves. Alternative fitness plots can be found in section 3 of the on‐line supporting material. We can see that each *orf*Δ curve fit represents the repeat level estimates well as each *orf*Δ level (red) curve lies in the region where most repeat level (black) curves are found. Sharing information between *orf*Δs will also affect each *orf*Δ curve fit, increasing the probability that the *orf*Δ level parameters are closer to the population parameters. Comparing Figs 2(a), 2(b) and 2(c) shows that the SHM captures heterogeneity at both the repeat and the *orf*Δ levels.

Fig. 2(d) demonstrates the hierarchy of information about the logistic model parameter *K* generated by the SHM for the *rad*50Δ control mutant strain (variation decreases going from population level down to repeat level). Fig. 2(d) also shows that the posterior distribution for *K* is much more peaked than the prior, demonstrating that we have learned about the distribution of both the population and the *orf*Δ parameters. Learning more about the repeat level parameters reduces the variance of our *orf*Δ level estimates. The posterior for the first time course repeat $K_{clm}$‐parameter shows exactly how much uncertainty there is for this particular repeat in terms of carrying capacity *K*.

#### 4.1.1 Fitness plots

Fitness plots are used to show which *orf*Δs show evidence of genetic interaction. The plots are typically mean *orf*Δ fitnesses for query strains against the corresponding control strains.

**Figure 3:**
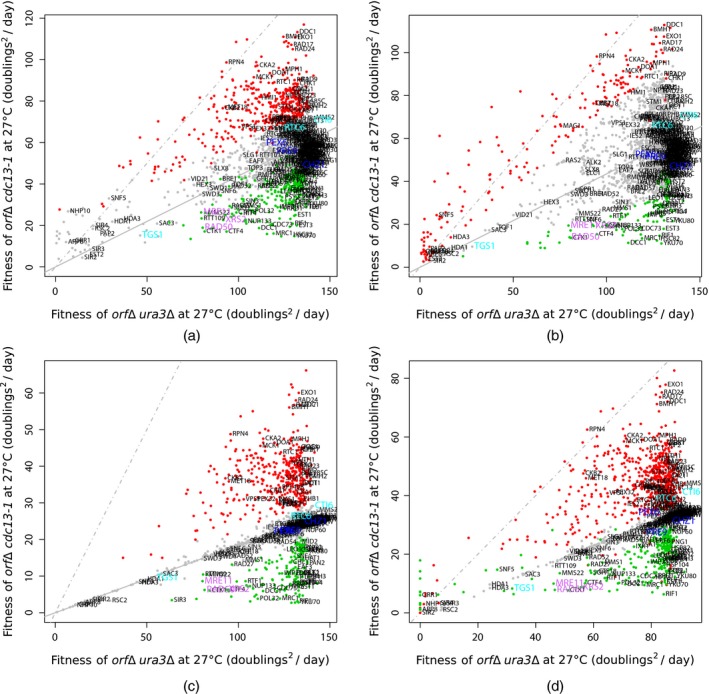
Fitness plots comparing mean fitnesses $\left(F=D_{R}D_{P}\right)$ for each *orf*Δ in a query and control screen (*orf*Δs significantly suppressing or enhancing the *cdc13‐1* fitness defect are highlighted in red and green respectively): (a) non‐Bayesian, non‐hierarchical fitness plot, based on Table S6 from Addinall *et al*. (2011); (b) non‐Bayesian, hierarchical fitness plot, from fitting the REM to data in Table S6 in Addinall *et al*. (2011); (c) IHM fitness plot; (d) JHM fitness plot (*orf*Δs are classified as suppressors or enhancers on the basis of an analysis of growth parameter *r*: some strains are fitter in the query experiment than predicted on the basis of the control but are classified as enhancers); (a), (b) significant interactors are classified as those with false discovery rate corrected *p*‐values less than 0.05; (c), (d) significant interactors have posterior probability $\delta_{l}>0.5$ (labelled genes are annotated with gene ontology terms from Table 2, ‘telomere maintenance’, ‘aging’, ‘response to DNA damage stimulus’ or ‘peroxisomal organization’, as well as genes identified as interactions by using the JHM by considering *K* (Fig. 4) (blue) or by considering *r* (cyan) and the MRX complex genes (pink) (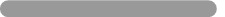, line of equal fitness; 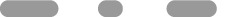, linear model fit)

Fig. 3(a) is a fitness plot from Addinall *et al*. (2011) where growth curves and evidence for genetic interaction are modelled by using the frequentist, non‐hierarchical methodology that was discussed in Section 2.2. Fig. 3(b) is a fitness plot for the frequentist hierarchical approach REM, described in equation (6), applied to the logistic growth parameter estimates that were used in Addinall *et al*. (2011). The number of genes identified as interacting with *cdc13‐1* by Addinall *et al*. (2011) and by the REM are 715 and 315 respectively (Table 2). The REM has highlighted many strains which have low fitness. To fit a linear model to the fitness data and to interpret results in terms of the multiplicative model we apply a log‐transformation to the fitnesses, thereby affecting the distribution of *orf*Δ level variation.

**Table 2:** Number of genes interacting with *cdc13‐1* at $27\;\;{}^{\circ }$C identified by using each of four approaches: Addinall *et al*. (2011) and the REM, IHM and JHM^a^

																		
*Method*	*Suppressors*	*Enhancers*	*Hits*	*Suppressors (half‐data)*	*Enhancers (half‐data)*	*Hits (half‐data)*	*Telomere maintenance* (*N*=33)	*p‐value*	*q‐value*	*Aging* (*N*=58)	*p‐value*	*q‐value*	*Response to DNA damage stimulus* (*N*=58)	*p‐value*	*q‐value*	*Peroxisome organization* (*N*=180)	*p‐value*	*q‐value*
Addinall *et al*. (2011)	419	296	715	263	192	455	18	$1.52\times 10^{-6}$	0.0376	16	$4.32\times 10^{-5}$	0.1863	69	$9.28\times 10^{-12}$	$8.14\times 10^{-10}$	13	0.225	0.468
REM	184	131	315	103	86	189	11	$2.37\times 10^{-5}$	0.0136	10	0.0004	0.0824	49	$7.40\times 10^{-16}$	$1.73\times 10^{-13}$	3	0.855	0.914
IHM	404	172	576	252	113	365	14	$6.57\times 10^{-5}$	0.0051	16	0.0015	0.0445	55	$4.60\times 10^{-9}$	$3.41\times 10^{-7}$	10	0.318	0.524
JHM	665	274	939	475	177	601	18	$8.22\times 10^{-5}$	0.0155	21	0.0015	0.0986	76	$3.52\times 10^{-9}$	$1.99\times 10^{-7}$	24	0.002	0.019

a Number of genes classified (or annotated) with four example gene ontology terms (telomere maintenance, aging, response to DNA damage stimulus and peroxisome organization) are also listed. For the approach of Addinall *et al*. (2011) and REM, significant interactors are classified as those with false discovery rate corrected *p*‐values (or *q*‐values) less than 0.05. The label ‘half‐data’ denotes analyses where only half of the available experimental observations are used.

The REM accounts for between‐subject variation and allows for the estimation of a query mutation and *orf*Δ effect to be made simultaneously, unlike the model that was presented by Addinall *et al*. (2011). Owing to the limitations of the frequentist hierarchical modelling framework, the REM model assumes equal variances for all *orf*Δs and incorrectly describes *orf*Δ level variation as log‐normal: assumptions that are not necessary in our new Bayesian approaches.

### 4.2 Application of the two‐stage modelling procedure to a suppressor–enhancer data set

Fig. 3(c) is an IHM fitness plot with *orf*Δ level fitness measures generated by using the new Bayesian two‐stage methodology with fitness in terms of $D_{R}D_{P}$. 576 genes are identified by the IHM as genetic interactions (Table 2). Logistic parameter posterior means are used to generate fitness measures. For a gene *l* from the gene deletion library, $\text{exp}(Z_{l})$ is the fitness for the control and $\text{exp}(\alpha_{1}+Z_{l}+\delta_{l}\gamma_{c,\;l})$ for the query. Similarly to Figs 3(a) and 3(b), Fig. 3(c) shows how the majority of control strains are more fitted than their query strain counterparts, with a mean fitted line lying below the line of equal fitness. Comparing the fitted lines in Figs 3(a) and 3(b) with Fig. 3(c) the IHM shows that the largest deviation between the fitted line and the line of equal fitness is largely due to the difference in *P* estimated with the SHM for the control and query data sets being scaled out by the parameter $\alpha_{1}$. If we fix *P* in our Bayesian models, as in the frequentist approach, genetic interactions identified are similar, but we then have the problem of choosing *P*. We recommend estimating *P* simultaneously with the other model parameters because, if the choice of *P* is not close to the true value, growth rate *r* estimates must compensate and do not give accurate estimates for time courses with low carrying capacity *K*.

It can be seen that many of the interacting *orf*Δs have large deviations from the genetic independence line. This is because of the indicator variable in the model, used to describe genetic interaction. When there is enough evidence for interaction the binary variable is set to 1; otherwise it is set to 0. It is interesting to note that non‐significant *orf*Δs, which are marked by grey points, lie among some of the significant strains. Many such points have high variance and we are therefore less confident that these interact with the query mutation. This feature of our new approach is an improvement over that presented in Addinall *et al*. (2011), which always shows evidence for an epistatic effect, for a given number of replicates, when the mean distance from the genetic independence line is large, regardless of actual strain fitness variability.

### 4.3 Application of the joint hierarchical model to a suppressor–enhancer data set

Fig. 3(d) is a JHM $D_{R}D_{P}$ fitness plot using the new, unified Bayesian methodology. 939 genes are identified by the JHM as genetic interactions (Table 2). Posterior means of model parameters are used to obtain the following fitness measures. For a gene *l* from the gene deletion library, $\left(\text{exp}\left(K_{l}^{o}\right),\text{exp}\left(r_{l}^{o}\right)\right)$ are used to evaluate the fitness for the control and $\left(\text{exp}\left(\alpha_{1}+K_{l}^{o}+\delta_{l}\gamma_{c,\;l}\right),\text{exp}\left(\beta_{1}+r_{l}^{o}+\delta_{l}\omega_{c,\;l}\right)\right)$ for the query.

Instead of producing a fitness plot in terms of $D_{R}D_{P}$, it can also be useful to analyse carrying capacity *K* and growth rate *r* fitness plots as, in the JHM, evidence for genetic interaction comes from both of these parameters simultaneously. Fitness plots in terms of logistic growth parameters are useful for identifying some unusual characteristics of *orf*Δs. For example, an *orf*Δ may be defined as a suppressor in terms of *K* but an enhancer in terms of *r*. To enable direct comparison with the analyses of Addinall *et al*. (2011). we generated a $D_{R}D_{P}$ fitness plot: Fig. 3(d).

### 4.4 Comparison with previous analysis

#### 4.4.1 Significant genetic interactions

Of the genes identified as interacting with *cdc13‐1* some are identified consistently across all four approaches (215 out of 1038; Table 3, part (a)). Of the hits identified by the JHM (939), the majority (639) are common with those in the previously published approach of Addinall *et al*. (2011). However, 231 of 939 are uniquely identified by the JHM and could be interesting candidates for further study.

**Table 3:** Genes interacting with *cdc13‐1* and gene ontology terms overrepresented in the list of interactions according to each approach^a^

		*REM:0*		*REM:1*	
		*Add:0*	*Add:1*	*Add:0*	*Add:1*
*(a)*					
IHM:0	JHM:0	3097	54	31	10
	JHM:1	231	78	29	29
IHM:1	JHM:0	1	2	1	0
	JHM:1	30	327	0	215
*(b)*					
IHM:0	JHM:0	5813	21	58	7
	JHM:1	46	8	6	10
IHM:1	JHM:0	20	15	3	12
	JHM:1	13	54	2	147

a Panel (a), number of genes identified for each approach (Addinall *et al*. (2011), REM, IHM and JHM) and the overlap between the approaches. 4135 genes from the *S. cerevisiae* single‐deletion library are considered. Panel (b), number of gene ontology terms identified for each approach and the overlap between the approaches. 6107 *S. cerevisiae* gene ontology terms were available.

**Figure 4:**
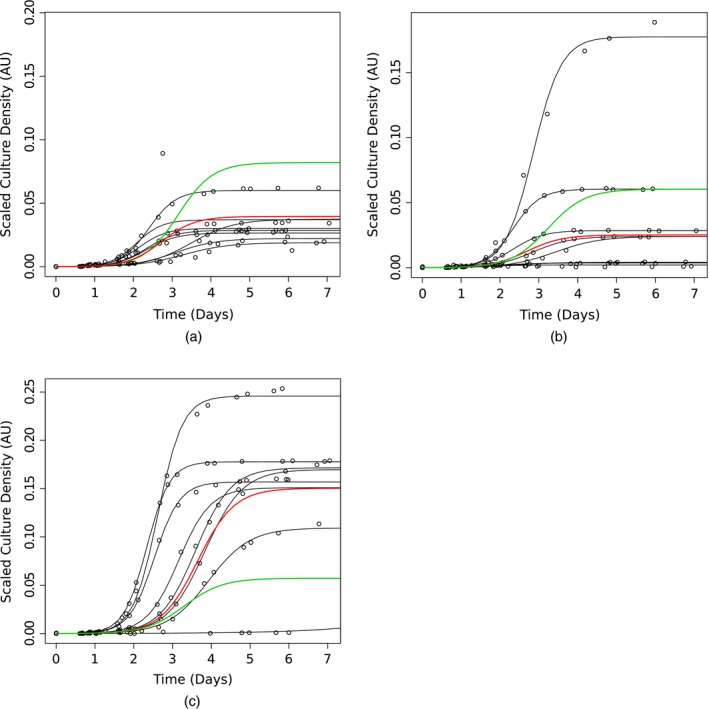
Hierarchy of growth curve model fits for the JHM for some example genotypes (JHM data for *orf*Δ repeats, with fitted curves overlaid in black for repeat level parameters, red for the *orf*Δ level query parameter fit and green for the expected *orf*Δ level query parameter fit with no genetic interaction): (a) JHM scatter plot for eight *chz1*Δ *cdc13‐1* repeats; (b) JHM scatter plot for eight *pre9*Δ *cdc13‐1* repeats; (c) JHM scatter plot for eight *pex6*Δ *cdc13‐1* repeats

To examine the evidence for some interactions uniquely identified by the JHM in more detail we compared the growth curves for three examples from the group of interactions identified only by the JHM. These examples (*chz*1Δ, *pre*9Δ and *pex*6Δ) are genetic interactions which can be identified in terms of carrying capacity *K*, but not in terms of growth rate *r* (which is a unique feature of the JHM; Fig. 4). By observing the difference between the fitted growth curve (red) and the expected growth curve, given no interaction (green) in Figs 4(a), 4(b) and 4(c) we test for genetic interaction. Since the expected growth curves in the absence of genetic interaction are not representative of either the data or the fitted curves on the repeat and *orf*Δ level, there is evidence for genetic interaction.

We chose a prior for the probability *p* of a gene interacting with the background mutation as 0.05, and we explore the effect of alternative choices below. We therefore expected to find 215 genes interacting. Using the Bayesian models, for which a prior is applicable (the IHM and JHM), we find more genes than expected (576 and 939 interactions respectively; Table 2), demonstrating that this data set is sufficiently information rich to overcome prior expectations. The JHM identifies the highest proportion of genes as hits out of all the methods considered, particularly identifying suppressors of *cdc13‐1* (Table 2). In fact, the JHM identifies more hits than the approach, of Addinall *et al*. (2011) even when constrained to using only half of the available data. An important advantage of our new Bayesian approaches is that we no longer have to choose a *q*‐value threshold. For the approach of Addinall *et al*. (2011) to have similar numbers of interactions to that of the JHM, a less stringent *q*‐value threshold would have to be justified *a posteriori* by the experimenter.

#### 4.4.2 Previously known genetic interactions

To compare the quality of our new, Bayesian hierarchical models with existing frequentist alternatives, we examined the lists of genetic interactions that were identified by all the methods discussed and presented here. Comparing results with expected or previously known lists of interactions from the relevant literature, we find that genes coding for the MRX complex (MRE11, RAD50 and XRS2), which are known to interact subtly with *cdc13‐1* (Foster *et al*., 2006), are identified by all four approaches considered and can be seen in a similar position on all four fitness plots (Figs 3(a), 3(b), 3(c) and 3(d)).

By observing the genes labelled in Figs 3(a) and 3(b) we can see that the frequentist approaches cannot identify many of the interesting genes identified by the JHM as these methods cannot detect interactions for genes that are close to the genetic independence line. It seems likely that the JHM has extracted more information from deletion strain fitnesses observed with high variability than the approach of Addinall *et al*. (2011) by sharing more information between levels of the hierarchy, consequently improving our ability to identify interactions for genes that are found closer to the line of genetic independence (subtle interactions). CTI6, RTC6 and TGS1 are three examples of subtle interactors identified only by the JHM (interaction in terms of *r* but not *K*) which all have previously known telomere‐related functions (Franke *et al*., 2008; Keogh *et al*., 2005; Addinall *et al*., 2008).

We tested the biological relevance of results from the various approaches by carrying out unbiased gene ontology (GO) term enrichment analyses on the hits (lists of genes classified as having a significant interaction with *cdc13‐1*) using the bioconductoR package GOstats (Falcon and Gentleman, 2007) (see section 2 of the on‐line supporting materials). As an example, fitness plots with genes co‐annotated with the ‘telomere maintenance’ highlighted can be seen in section 3 of the on‐line supporting materials.

Extracts from the list of top interactions identified by both the IHM and the JHM are provided in section 4 of the on‐line supporting materials. Files including the full lists of genetic interactions for the IHM and JHM are also provided (http://research.ncl.ac.uk/qfa/HeydariQFABayes/). Since we can use the JHM to identify interactions in terms of both *K* and *r* simultaneously, it is useful to order lists of suppressors and enhancers in terms of *K* and *r* as well as a fitness measure such as $D_{R}D_{P}$ for reviewing the results; see section 5 of the on‐line supporting materials.

All methods identify a large proportion of the genes in the yeast genome annotated with the GO terms telomere maintenance and ‘response to DNA damage stimulus’ (see Table 2 and the on‐line supporting materials), which were the targets of the original screen, demonstrating that they all correctly identify previously known hits of biological relevance. Interestingly, the JHM identifies many more genes annotated with the ‘aging’ GO term, which we also expect to be related to telomere biology (though the role of telomeres in aging remains controversial) suggesting that the JHM is identifying novel relevant interactions that were not previously identified by the screen of Addinall *et al*. (2011) (see Table 2). Similarly, the JHM identifies a much larger proportion of the PEX ‘peroxisomal’ complex (included in GO term ‘peroxisome organization’) as interacting with *cdc13‐1* (see Table 2) including all of those identified in Addinall *et al*. (2011). Many of the PEX genes show large variation in both *K* and *r*; an example can be seen in Fig. 4(c) for *pex6*Δ. Members of the PEX complex cluster tightly, above the fitted line in the fitness plot Fig. 3(d) (fitness plots with highlighted genes for GO terms in Table 2 are given in section 3 of the on‐line supporting materials), demonstrating that, although these functionally related genes are not strong interactors, the same behaviour is reproduced independently by multiple members of a known functional complex, suggesting that the predicted interactions are real. The results of tests for significant overrepresentation of all GO terms can be found on line: http://research.ncl.ac.uk/qfa/HeydariQFABayes/.

Overall, within the lists of genes identified as interacting with *cdc13‐1* by the approach of Addinall *et al*. (2011) and the REM, IHM and JHM, 274, 245, 266 and 286 GO terms were significantly overrepresented respectively (out of 6235 possible GO terms; see Table 3, part (b)). 147 were common to all approaches and examples from the group of GO terms overrepresented in the JHM analysis and not in the Addinall *et al*. (2011) analysis seem internally consistent (e.g. the peroxisome organization GO term) and consistent with the biological target of the screen, telomere biology (significant GO terms for genes identified only by the JHM are also included in the spreadsheet document provided in the on‐line supporting materials).

**Figure 5:**
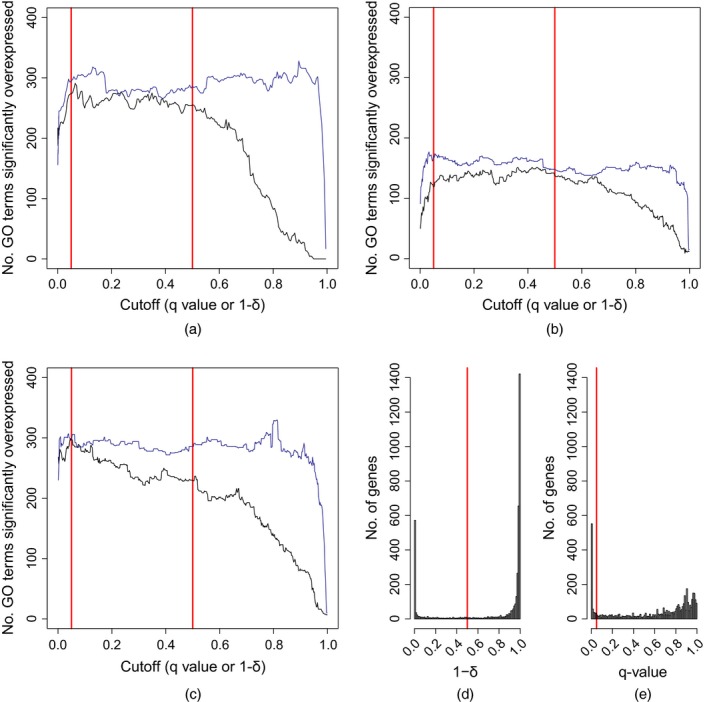
Sensitivity to significance thresholds: (a)–(c) comparison of the number of significantly overexpressed GO terms expressed in lists of significant interactors found by using the method of Addinall *et al*. (2011) (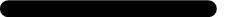), and by using the JHM (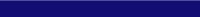), and frequency histograms showing distributions of classifier values after looking for genes interacting with *cdc13‐1* at 27 ${}^{\circ }$C by using (d) the JHM or (e) the method of Addinall *et al*. (2011)

A major advantage of the Bayesian approaches that are presented here over that of Addinall *et al*. (2011) is the measure that is used for classifying significant interactions. Classifying interactions with a posterior estimate for $\delta_{l}$ (the probability that an interaction exists) greater than 0.5 as significant is less arbitrary than the traditional frequentist approach of classifying interactions with *p*‐values less than 0.05 as significant. Examining how the number of over‐represented GO terms found in lists of interactors varies with the classification threshold shows that the Bayesian JHM approach is also less sensitive to the precise threshold values that are used. Fig. 5 shows that the number of overexpressed GO terms found among hits is relatively stable in the region of $\delta_{l}=0.5$ for the JHM compared with the equivalent number in the region of *q*=0.05 for the approach of Addinall *et al*. (2011). Significantly overexpressed GO terms were identified by using the hyperGTest function in the GOstats R package. Note that the values that are used to classify whether a gene interacts with *cdc13‐1* at 27 ${}^{\circ }C$ (the *q*‐value and *δ*; red vertical lines as presented in Section 4.4) are not directly comparable; however, the full range of possible cut‐offs for both values are plotted. In particular, using the frequentist approach of Addinall *et al*. (2011), the number of overexpressed GO terms falls rapidly where *q*<0.05. We tested whether this observation depended on our choice of the parameter *p*, which represents our prior expectation of the proportion of genes interacting with the query, by generating similar sensitivity plots for *p* between 0.01 and 0.2 (section 6 of the on‐line supporting materials). We observed similar profiles of overexpressed GO terms for all values of *p* tested.

Comparing the genetic interaction strengths generated by the Bayesian hierarchical models and frequentist analysis, we find that the results are largely similar (section 6 of the on‐line supporting materials); however, the GO term analysis described above suggests that the differences are important.

The results of a simulation study comparing the sensitivity and specificity of the approach of Addinall *et al*. (2011), the REM, the SHM and the JHM are summarized in section 8 of the supplementary materials. We find that the JHM correctly identified a higher proportion of ‘true’ interactions in a synthetic data set than did the approach of Addinall *et al*. (2011)

#### 4.4.3 Hierarchy and model parameters

The hierarchical structure and model choices that were included in the Bayesian JHM and IHM are derived from the known experimental structure of QFA. Different levels of variation for different *orf*Δs are expected and can be observed by comparing distributions of frequentist estimates or by visual inspection of yeast culture images. The direct relationship between experimental and model structure, together with the richness of detail and number of replicates included in QFA experimental design, reassures us that overfitting is not an issue in this analysis. For the *ura3*Δ 27 ${}^{\circ }$C and *cdc13‐1* 27 ${}^{\circ }$C experiment with about 4294 *orf*Δs there are roughly 1.25 times the number of parameters in the JHM (of the order of 200000) compared with the two‐stage REM approach (of the order of 160000) but when compared with the large number of pairs of data points (of the order of 830000) there are sufficient degrees of freedom to justify our proposed Bayesian models.

#### 4.4.4 Computing requirements

Our Bayesian hierarchical models require significant computational time. As expected, the mixing of chains in our models is weakest at population level parameters such as $K_{p}$ and $\alpha_{c}$. For the *ura3*Δ 27 ${}^{\circ }$C and *cdc13‐1* 27 ${}^{\circ }$C data set, running with a Markov chain Monte Carlo burn‐in of 800000 updates, followed by generating 1000 samples thinned by a factor of 100, the JHM takes about 4 weeks to converge and produce a sufficiently large sample. The two‐stage Bayesian approach takes 1 week (with the IHM part taking about 1 day), whereas the REM takes about 3 days and the approach of Addinall *et al*. (2011) takes about 3 h. A QFA experiment can take over a month from start to finish and so the analysis time is acceptable in comparison with the time taken for the creation of the data set but is still a notable inconvenience. We expect that, with further research effort, the computational time can be decreased by using an improved inference scheme and that inference for the JHM could be completed in less than a week without parallelization.

## 5 Discussion

We have joined a hierarchical model of microbial growth with a model for genetic interaction to learn about strain fitnesses, evidence for genetic interaction and interaction strengths simultaneously. By introducing Bayesian methodology to QFA we have been able to model the hierarchical nature of the experiment and to expand the multiplicative model for genetic interaction to incorporate many sources of variation that previously had to be ignored.

We propose two new Bayesian hierarchical models to replace the current statistical analysis for identifying genetic interactions within a QFA screen comparison. The two‐stage approach fits the SHM followed by the IHM, with univariate point estimate fitness definitions generated as an intermediate step. The two‐stage approach can therefore be regarded as a Bayesian hierarchical version of the approach of Addinall *et al*. (2011). In contrast, the one‐stage approach fits the JHM, which does not require a separate definition of fitness, allowing interaction to be identified by either growth rate (logistic parameter *r*) or final biomass achievable (logistic parameter *K*) by a given genotype. Our one‐stage approach is a new method for detecting genetic interaction that further develops the interpretation of epistasis within QFA screens.

We present a hierarchical, frequentist approach using random effects, namely the REM, in an attempt to improve on the approach of Addinall *et al*. (2011). Owing to the lack of flexibility in modelling assumptions allowable, the REM is unsuitable for modelling the distribution of *orf*Δ level variation or for simultaneously modelling genetic interaction and logistic growth curves.

The data from which logistic parameter estimates are derived during QFA are the result of a technically challenging, high throughput experimental procedure with a diverse range of possible technical errors. Our Bayesian hierarchical models allow us the flexibility to make distributional assumptions that more closely match the data. This allows us to switch between modelling parameter uncertainty with the normal, log‐normal and Student *t*‐distribution where appropriate.

QFA experimental design is intrinsically multilevel and is therefore more closely modelled by our hierarchical scheme. Consequently the JHM and IHM capture sources of variation that were not considered by Addinall *et al*. (2011). By sharing information across levels in the hierarchy, our models have allowed us to learn more about *orf*Δs with weaker genetic interaction. Our more flexible model of variance also avoids misclassification of individual genotypes with high variance as having significant interactions. Without fully accounting for the variation that is described in the Bayesian hierarchical models, the previous approach of Addinall *et al*. (2011) may have relatively poor power to detect subtle interactions, obscuring potentially novel observations.

Many subtle, interesting genetic interactions may remain to be identified in the data from the QFA experiments that we reanalyse in this paper. The JHM is better able to identify subtle interactions. For example, strains with little evidence for interaction with a background mutation in terms of growth rate but with strong evidence of interaction in terms of carrying capacity are sometimes classified as interactors by using the JHM (see Fig. 4). In our two‐stage approaches, univariate fitness measures such as $D_{R}D_{P}$ are used in the intermediate steps, occasionally causing interaction in terms of one parameter to be masked by the other.

As expected, many genes that were previously unidentified by Addinall *et al*. (2011) have been identified as showing evidence of interaction by using both of our Bayesian hierarchical modelling approaches. Genes which have been identified only by the JHM (see Fig. 3(d)), such as those showing interaction only in terms of *r*, are found to be related to telomere biology in the literature. Currently sufficient information is not available to identify the proportion of identified interactions that are true hits and so we use unbiased GO term enrichment analyses to confirm that the lists of genetic interactions closely reflect the true underlying biology. GO term annotations that are relevant to telomere biology are available for well‐studied genes in the current literature. Unsurprisingly all of the approaches considered closely reflect the most well‐known GO terms (see Table 2).

Computational time for the new Bayesian approach ranges from 1 to 4 weeks for one of the data sets that was presented in Addinall *et al*. (2011). This is of the same magnitude as the time taken to design and execute the experimental component of QFA (approximately 6 weeks).

Overall we recommend a JHM or ‘Bayesian QFA’ for analysis of current and future QFA data sets as it accounts for more sources of variation than the QFA methodology of Addinall *et al*. (2011). With the JHM we have outlined new genes with significant evidence of interaction in the *ura3* $\Delta \;27\;\;{}^{\circ }C$ and *cdc13‐1* $\;27\;\;{}^{\circ }C$ experiment. The new Bayesian hierarchical models that we present here will also be suitable for identifying new genes showing evidence of genetic interaction in backgrounds other than telomere activity. We hope that further reductionist laboratory work by experimental biologists will give additional insight into the mechanisms by which the new genes that we have uncovered interact with the telomere.

## Supporting information

‘Web‐based supporting materials for “Bayesian hierarchical modelling for inferring genetic interactions in yeast”’.
