# Supplementary material for: Bayesian hierarchical modelling for inferring genetic interactions in yeast
Source: J R Stat Soc Ser C Appl Stat. 2015 Oct 29;65(3):367–93. doi: 10.1111/rssc.12126 (PMC4843957; doi:10.1111/rssc.12126)
Supplement: Supplementary file 1 — ‘Web‐based supporting materials for “Bayesian hierarchical modelling for inferring genetic interactions in yeast”’. [file RSSC-65-367-s001.pdf]

Web-based supporting materials for "Bayesian hierarchical modelling for inferring genetic interactions in yeast" by Jonathan Heydari, Conor Lawless, David A. Lydall and Darren J. Wilkinson

1 Plate diagrams

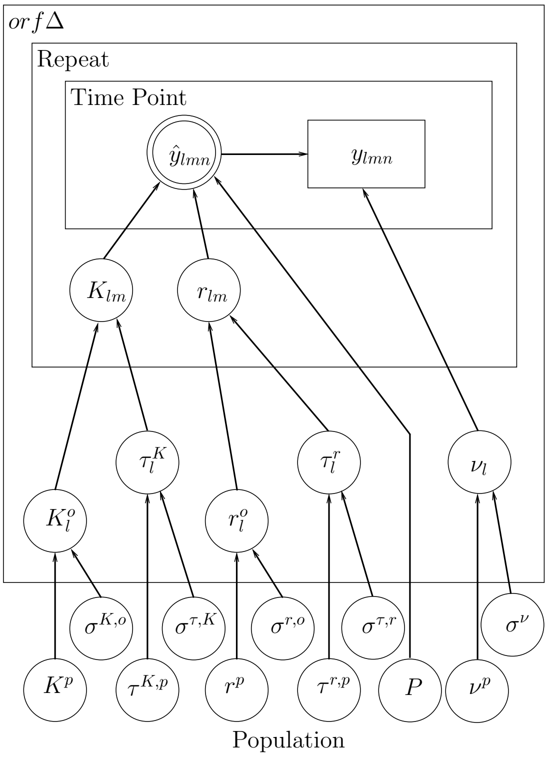

Figure 1.1: Plate diagram for the SHM, described in Section 3.1 of the main article. This figure shows the four levels of hierarchy in the SHM model, population, *orf*Δ (l), repeat (m) and time point (n). Prior hyperparameters for the population parameters are omitted. A circular node represents a parameter in the model. An arrow from a source node to a target node indicates that the source node parameter is a prior hyperparameter for the target node parameter. Each rectangular box corresponds to a level of the hierarchy. Nodes within multiple boxes are nested and their parameters are indexed by corresponding levels of the hierarchy. The node consisting of two concentric circles corresponds to our model's fitted values. The rectangular node represents the observed data.

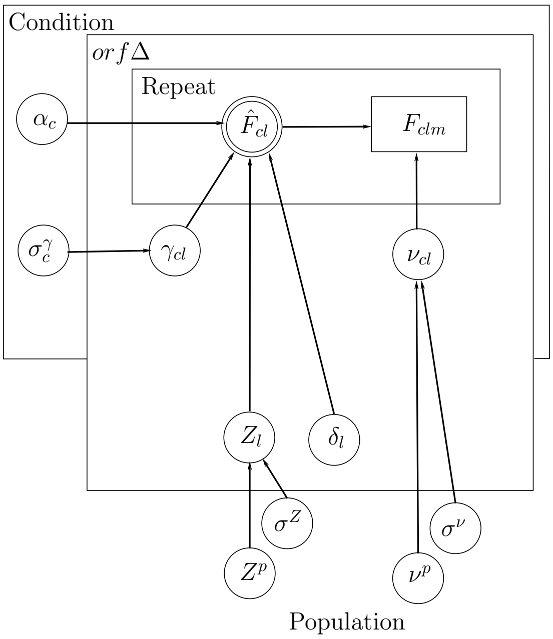

Figure 1.2: Plate diagram for the IHM, described in Section 3.2 of the main article. This figure shows the four levels of hierarchy in the IHM model: population, *orf*Δ (l), condition (c) and repeat (m). Prior hyperparameters for population parameters are omitted. Plate diagram notation as in Figure 1.1.

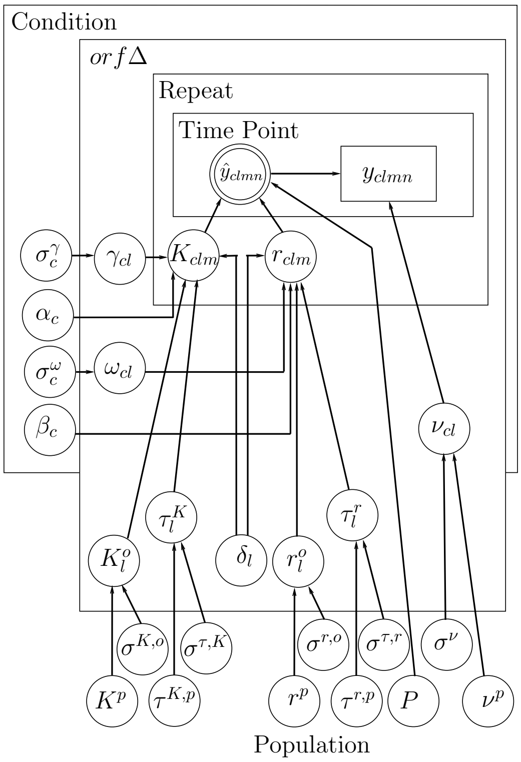

Figure 1.3: Plate diagram the JHM, described in Section 3.3 of the main article. This figure shows the five levels of hierarchy in the JHM model, population, *orf*Δ (l), condition (c), repeat (m) and time point (n). Prior hyperparameters for the population parameters are omitted. Plate diagram notation is given in Figure 1.1.

2 GO term enrichment analysis in R

```
source("http://bioconductor.org/biocLite.R")
biocLite("GOstats")
biocLite("org.Sc.sgd.db")
###################
library(GOstats) # GO testing tool package
library(org.Sc.sgd.db) # yeast gene annotation package
genes=read.table("sm_JHM_list.txt", header=T)
UNIVSTRIP=genes[,2]
genes<-as.vector(genes[genes[,3]>0.5,2])
genes<-unique(genes)
ensemblIDs=as.list(org.Sc.sgdPMID2ORF)
univ=unlist(ensemblIDs)
univ=univ[!is.na(univ)]
length(univ)
length(unique(univ))
univ=unique(univ)
all=as.vector(univ)
all=all[all%in%UNIVSTRIP]
length(all)
ontology=c("BP")
vec<-genes%in%univ
genes<-genes[vec]
params_temp=new("GOHyperGParams", geneIds=genes,
universeGeneIds=all,
 annotation="org.Sc.sgd.db", categoryName="GO",
 ontology=ontology, pvalueCutoff=1,
 testDirection = "over")
results=hyperGTest(params_temp)
results=summary(results)
results$qvalue<-p.adjust(results$Pvalue,method="BH")
```

3 Fitness plots with GO terms highlighted

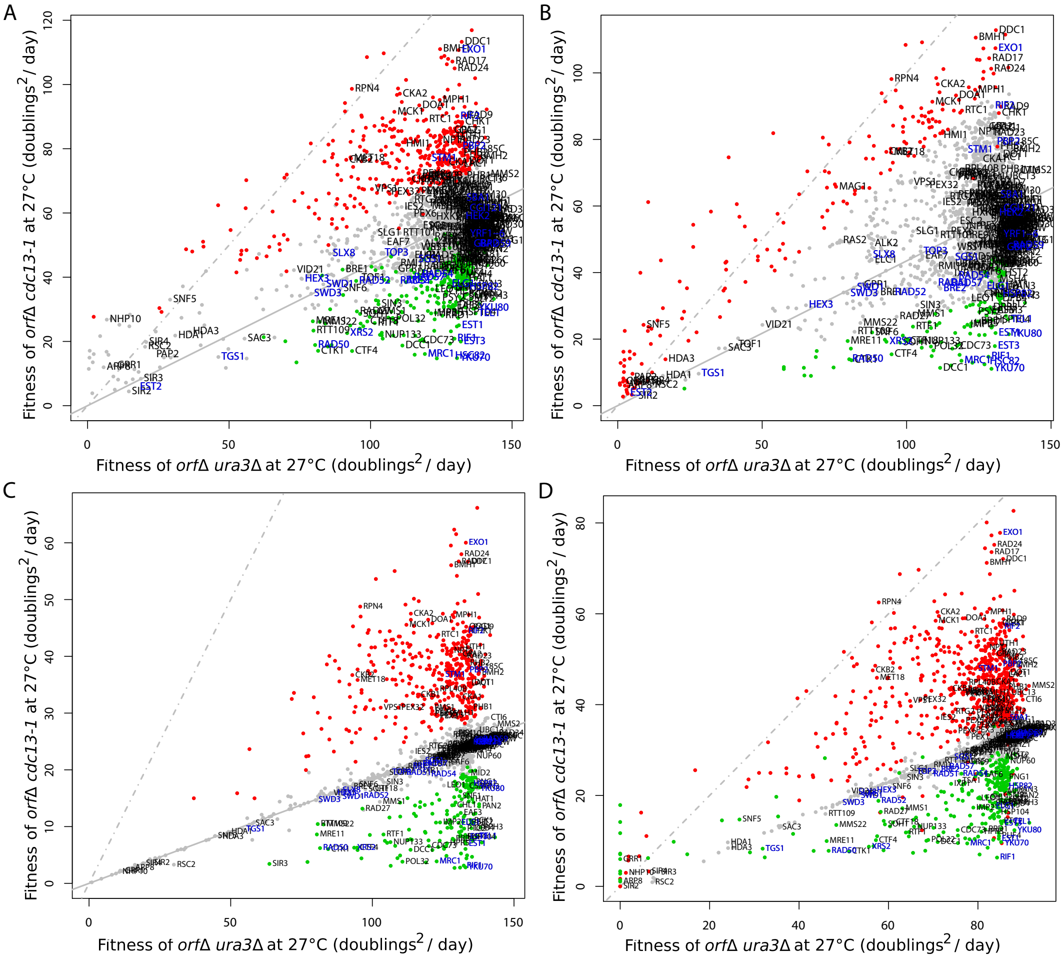

Figure 3.1: Alternative fitness plots with *orf*Δ posterior mean fitnesses. Text for the "telomere maintenance" GO term is highlighted in blue. A) Non-Bayesian, non-hierarchical fitness plot, based on Table S6 from Addinall et al. (2011) $(F = MDR \times MDP)$. B) Non-Bayesian, hierarchical fitness plot, from fitting REM to data in Table S6 from Addinall et al. (2011) $(F = MDR \times MDP)$. C) IHM fitness plot with *orf*Δ posterior mean fitness $(F = MDR \times MDP)$. D) JHM fitness plot with *orf*Δ posterior mean fitnesses. *orf*Δ strains are classified as being a suppressor or enhancer based on analysis of growth parameter r. Further explanation and notation for fitness plots are given in Figure 3 of the main article.

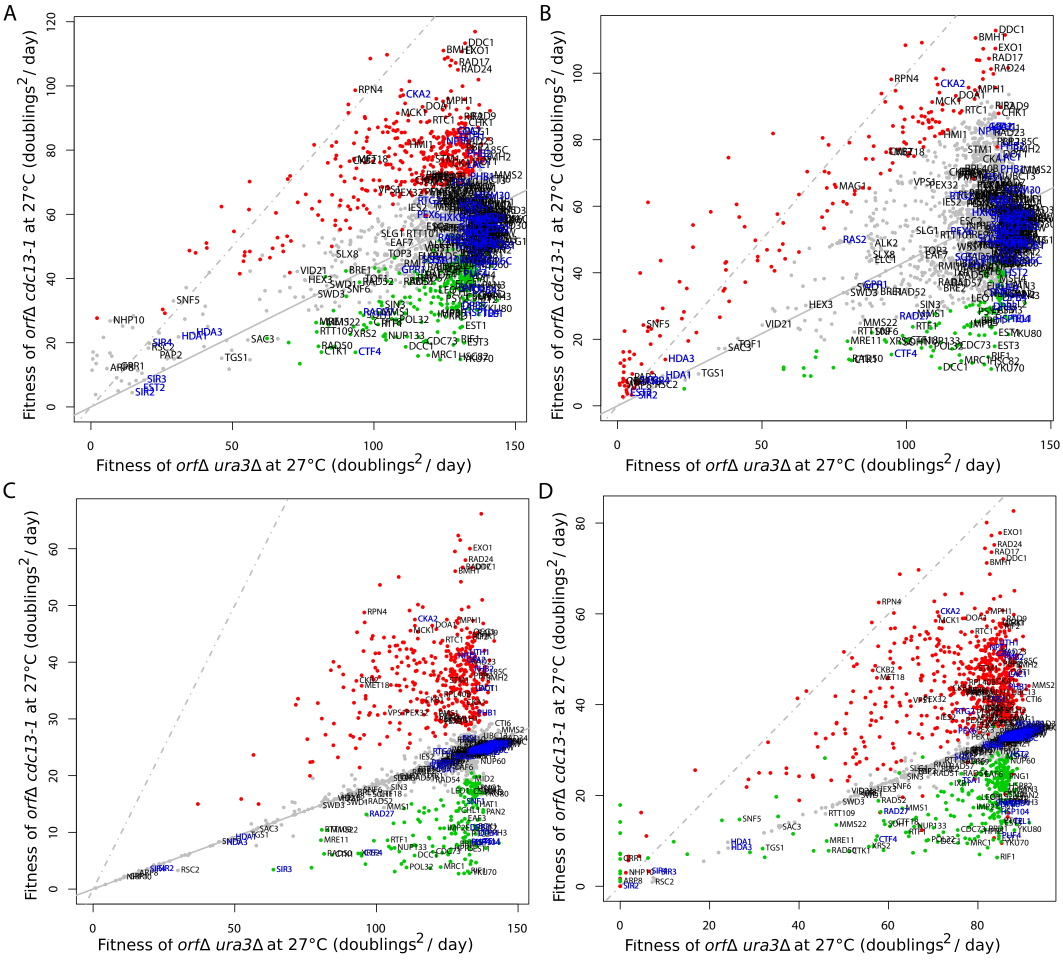

Figure 3.2: Alternative fitness plots with *orf*Δ posterior mean fitnesses. Text for the "ageing" GO term is highlighted in blue. A) Non-Bayesian, non-hierarchical fitness plot, based on Table S6 from Addinall et al. (2011) $(F = MDR \times MDP)$. B) Non-Bayesian, hierarchical fitness plot, from fitting REM to data in Table S6 from Addinall et al. (2011) $(F = MDR \times MDP)$. C) IHM fitness plot with *orf*Δ posterior mean fitness $(F = MDR \times MDP)$. D) JHM fitness plot with *orf*Δ posterior mean fitnesses. *orf*Δ strains are classified as being a suppressor or enhancer based on analysis of growth parameter r. Further explanation and notation for fitness plots are given in Figure 3 of the main article.

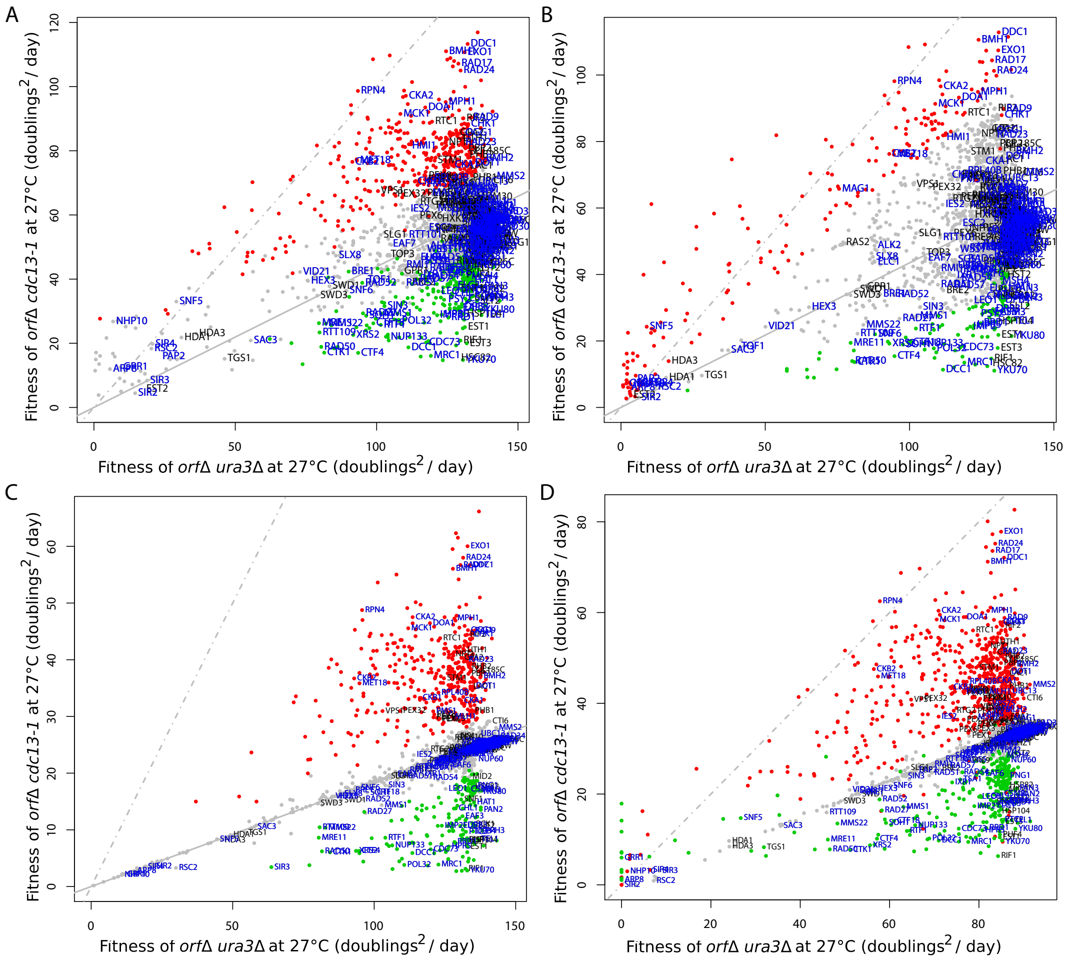

Figure 3.3: Alternative fitness plots with *orf*Δ posterior mean fitnesses. Text for the "response to DNA damage" GO term is highlighted in blue. A) Non-Bayesian, non-hierarchical fitness plot, based on Table S6 from Addinall et al. (2011) $(F = MDR \times MDP)$. B) Non-Bayesian, hierarchical fitness plot, from fitting REM to data in Table S6 from Addinall et al. (2011) $(F = MDR \times MDP)$. C) IHM fitness plot with *orf*Δ posterior mean fitness $(F = MDR \times MDP)$. D) JHM fitness plot with *orf*Δ posterior mean fitnesses. *orf*Δ strains are classified as being a suppressor or enhancer based on analysis of growth parameter r. Further explanation and notation for fitness plots are given in Figure 3 of the main article.

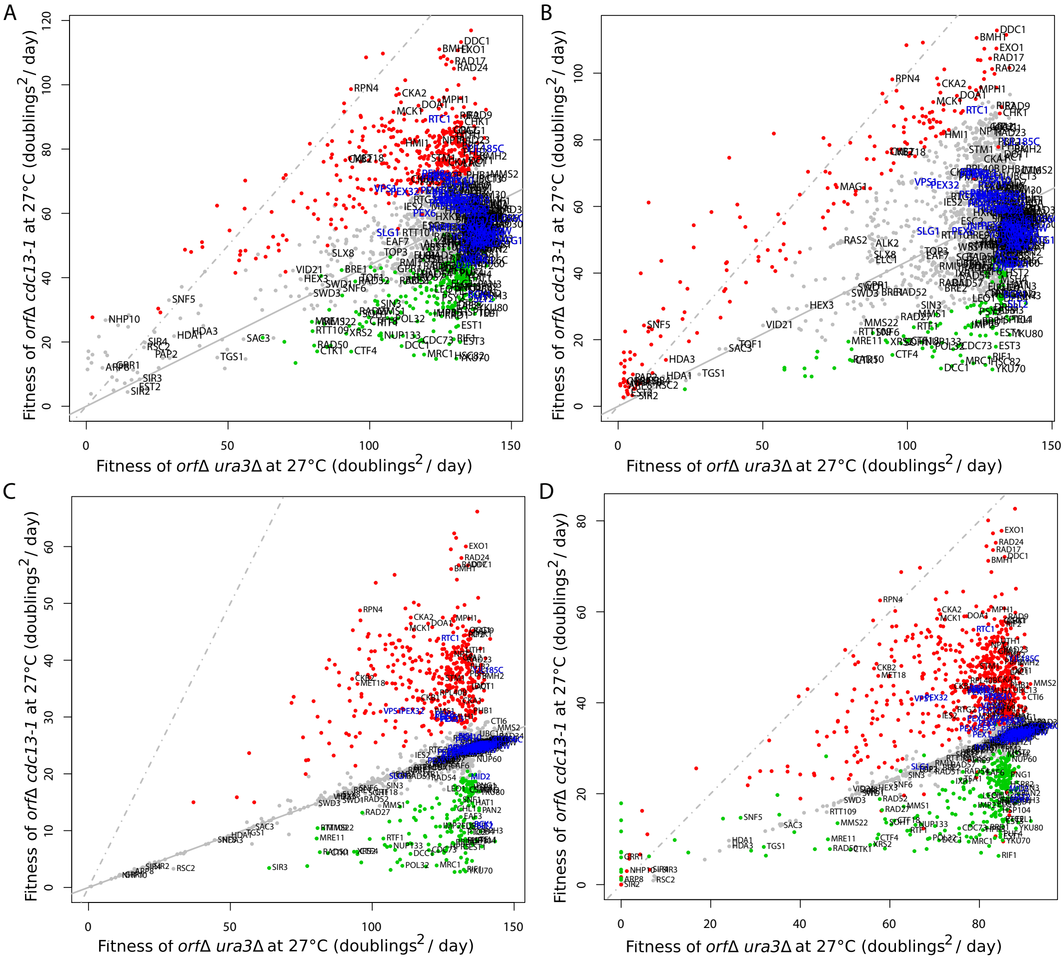

Figure 3.4: Alternative fitness plots with *orf*Δ posterior mean fitnesses. Text for the "peroxisomal organisation" GO term is highlighted in blue. A) Non-Bayesian, non-hierarchical fitness plot, based on Table S6 from Addinall et al. (2011) ($F = MDR \times MDP$). B) Non-Bayesian, hierarchical fitness plot, from fitting REM to data in Table S6 from Addinall et al. (2011) ($F = MDR \times MDP$). C) IHM fitness plot with *orf*Δ posterior mean fitness ($F = MDR \times MDP$). D) JHM fitness plot with *orf*Δ posterior mean fitnesses. *orf*Δ strains are classified as being a suppressor or enhancer based on analysis of growth parameter r. Further explanation and notation for fitness plots are given in Figure 3 of the main article.

4 Lists of top genetic interactions for IHM and JHM approaches

Type of Interaction	*Gene Name*	*Probability of Interaction δ_l*	*Strength of Interaction $e^{(\delta_l \gamma_l)}$*	*Position in Addinall (2011)*
Suppressor	IPK1	1.00	2.87	10
	LST4	1.00	2.77	13
	RPN4	1.00	2.76	17
	MTC5	1.00	2.66	20
	GTR1	1.00	2.64	38
	NMD2	1.00	2.62	3
	SAN1	1.00	2.62	16
	UPF3	1.00	2.58	21
	RPL37A	1.00	2.56	121
	NAM7	1.00	2.53	22
	RPP2B	1.00	2.52	120
	YNL226W	0.99	2.49	126
	YGL218W	1.00	2.46	250
	MEH1	1.00	2.45	45
	ARO2	1.00	2.45	68
	EXO1	1.00	2.45	1
	BUD27	1.00	2.43	46
	RAD24	1.00	2.39	4
	RPL16B	1.00	2.39	33
	RPL43A	1.00	2.39	150
Enhancer	MRC1	1.00	0.11	35
	YKU70	1.00	0.11	31
	STI1	1.00	0.11	42
	RIF1	1.00	0.13	36
	ELP3	1.00	0.16	82
	CLB5	1.00	0.17	58
	MRC1	1.00	0.17	63
	DPH2	1.00	0.18	24
	POL32	1.00	0.19	113
	MAK31	1.00	0.19	37
	SWM1	1.00	0.20	25
	LTE1	1.00	0.21	48
	MAK10	1.00	0.22	44
	ELP2	1.00	0.22	77
	PAT1	1.00	0.24	144
	DPH1	1.00	0.25	55
	SRB2	0.99	0.25	174
	THP2	1.00	0.26	67
	MFT1	1.00	0.26	52
	LSM6	0.97	0.26	389

A file containing the full list of genetic interactions is also provided in the on-line supporting materials.

Table 4.1: Sample of IHM top genetic interactions

Type of Interaction	*Gene Name*	*Probability of Interaction* δ_l	*Strength of Interaction* $e^{(\delta_l \gamma_l)}$	*Strength of Interaction* $e^{(\delta_l \omega_l)}$	*Strength of Interaction* $MDR \times MDP$	*Position in Addinall (2011)*
Suppressor in K	CSE2	1.00	490.51	0.48	11.71	838
	SGF29	1.00	273.69	0.68	14.16	580
	GSH1	1.00	78.79	0.92	17.89	281
	YMD8	1.00	59.31	0.65	7.05	2022
	YGL024W	1.00	28.13	1.18	13.33	151
	RPS9B	1.00	24.67	1.12	10.24	801
	GRR1	1.00	22.51	0.67	5.99	1992
Suppressor in r	BTS1	1.00	19.27	2.29	19.65	201
	IPK1	1.00	5.56	2.26	44.81	10
	NMD2	1.00	2.96	2.19	48.51	3
	SAN1	1.00	2.37	2.17	48.70	16
	LST4	1.00	5.79	2.14	44.14	13
	RPN4	1.00	8.00	2.12	40.46	17
	UPF3	1.00	3.16	2.07	45.25	21
Suppressor in $MDR \times MDP$	SAN1	1.00	2.37	2.17	48.70	16
	NMD2	1.00	2.96	2.19	48.51	3
	UPF3	1.00	3.16	2.07	45.25	21
	EXO1	1.00	2.89	2.06	45.04	1
	IPK1	1.00	5.56	2.26	44.81	10
	LST4	1.00	5.79	2.14	44.14	13
	NAM7	1.00	3.02	2.04	43.00	22
Enhancer in K	YKU70	1.00	0.01	1.09	−23.44	31
	STI1	1.00	0.01	1.20	−21.60	42
	RIF1	1.00	0.01	0.63	−26.17	36
	MRC1	1.00	0.01	0.83	−23.15	35
	MAK31	1.00	0.02	1.18	−18.19	37
	CLB5	1.00	0.02	0.87	−19.54	58
	MRC1	1.00	0.02	0.81	−20.40	63
Enhancer in r	PAT1	1.00	1.71	0.28	−18.30	144
	PUF4	1.00	2.00	0.31	−21.61	34
	YKU80	1.00	2.15	0.33	−21.68	32
	RTT103	1.00	2.54	0.34	−17.87	153
	LSM1	0.99	2.13	0.34	−16.20	101
	GIM3	0.99	0.93	0.35	−19.70	132
	INP52	0.96	0.86	0.36	−14.50	345
Enhancer in $MDR \times MDP$	RIF1	1.00	0.01	0.63	−26.17	36
	LTE1	1.00	0.06	0.40	−23.96	48
	YKU70	1.00	0.01	1.09	−23.44	31
	MRC1	1.00	0.01	0.83	−23.15	35
	DPH2	1.00	0.04	0.56	−23.11	24
	EST1	1.00	0.12	0.46	−22.20	5
	MAK10	1.00	0.04	0.59	−21.92	44

A file containing the full list of genetic interactions is also provided in the on-line supporting materials.

Table 4.2: Sample of JHM top genetic interactions

5 Alternative fitness plots for the JHM

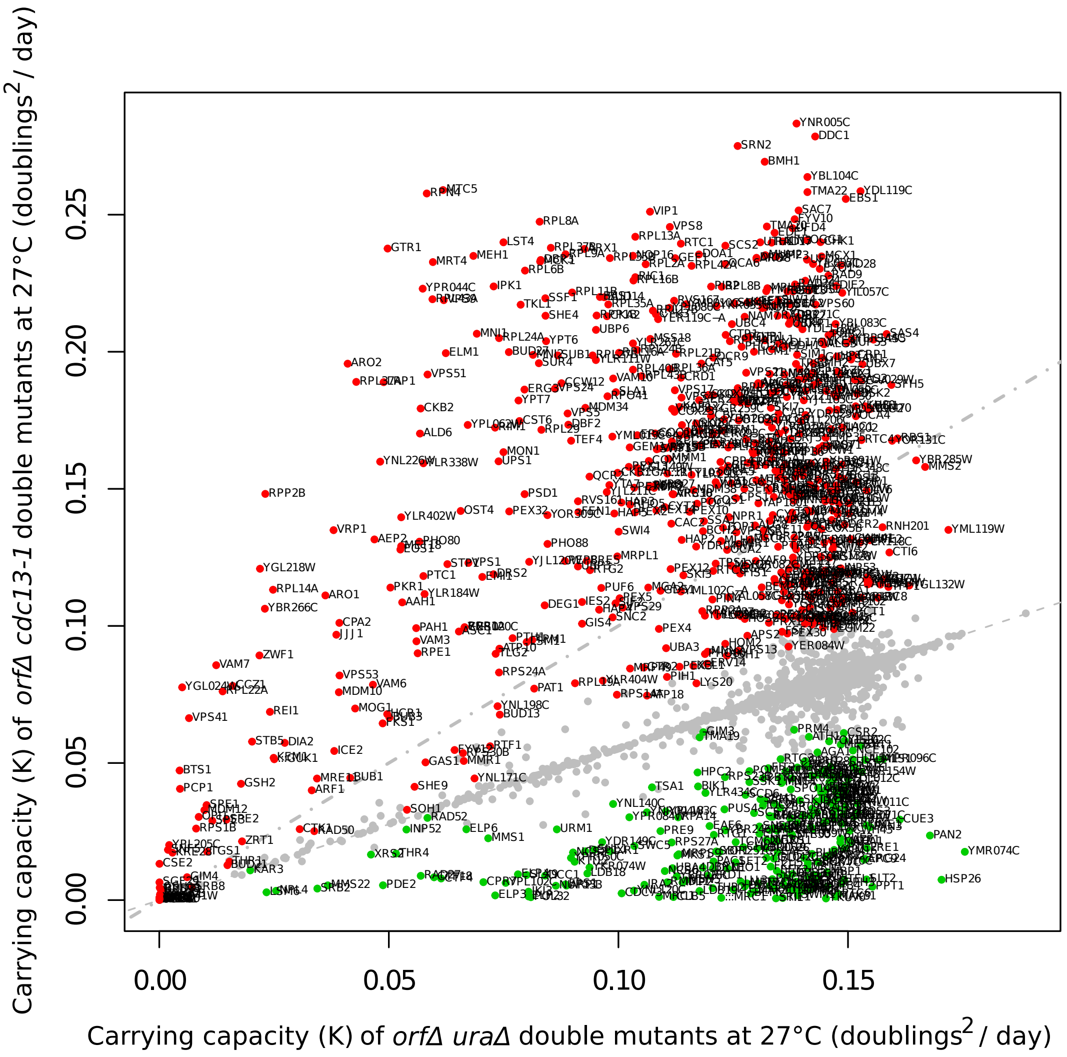

Figure 5.1: Joint hierarchical model (JHM) carrying capacity fitness plot with *orf*Δ posterior mean fitnesses. *orf*Δ strains are classified as being a suppressor or enhancer based on carrying capacity parameter K. Further explanation and notation for fitness plots are given in Figure 3 of the main article.

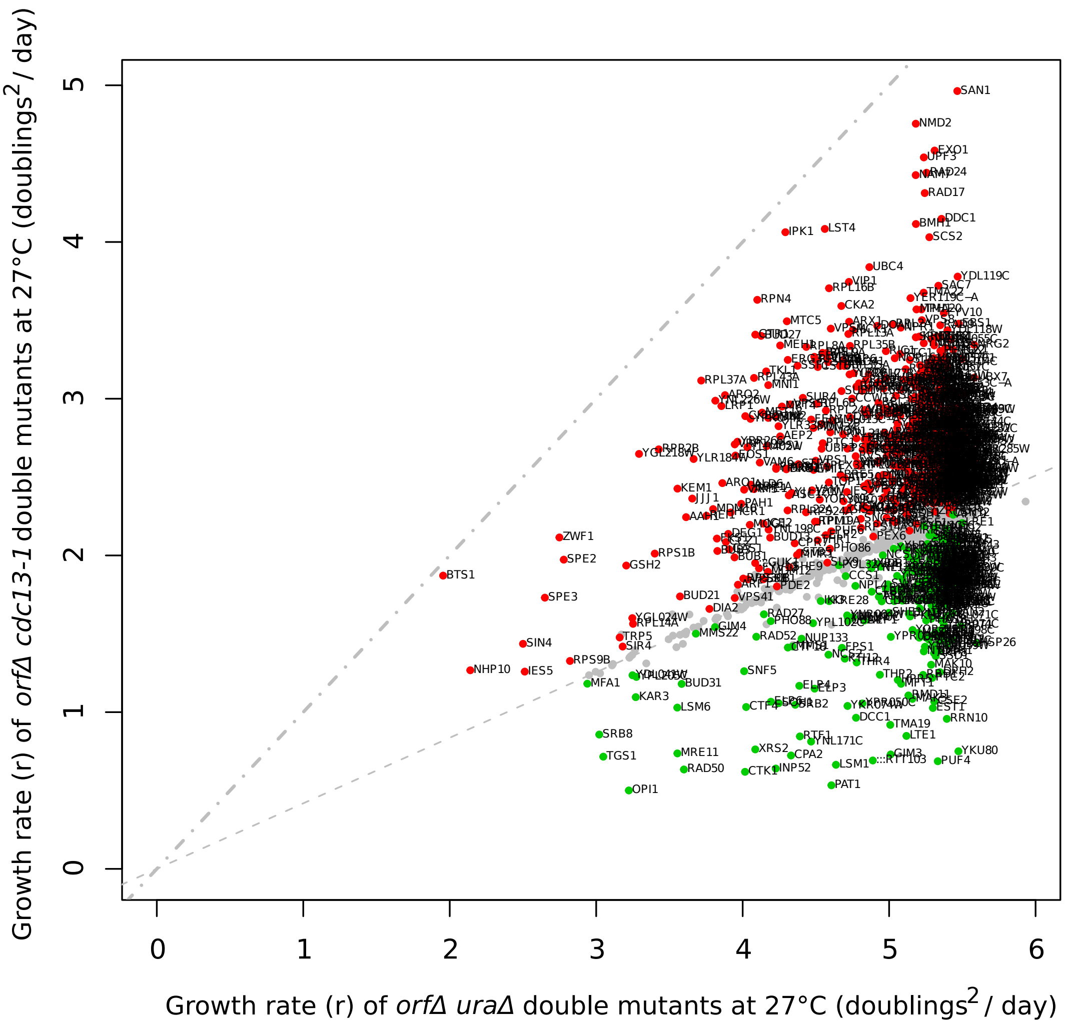

Figure 5.2: Joint hierarchical model (JHM) growth rate fitness plot with *orf*Δ posterior mean fitnesses. *orf*Δ strains are classified as being a suppressor or enhancer based on growth parameter r. Further explanation and notation for fitness plots are given in Figure 3 of the main article.

6 The effect of parameter p on specificity

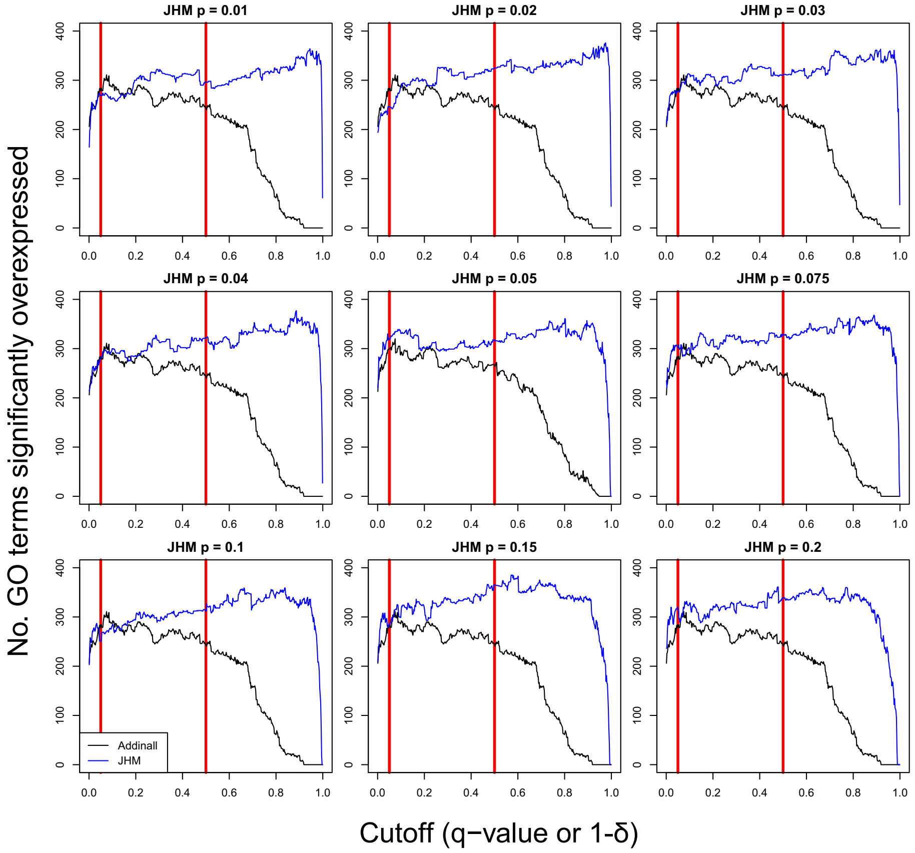

Figure 6.1: Comparison of the number of significantly over-expressed GO terms identified in lists of significant interactors found using the Addinall (2011) method and using the JHM. Significantly over-expressed GO terms were identified using the hyperGTest function in the GOstats R package. Note that the values used to classify whether a gene interacts with *cdc13-1* at 27C (q-value and δ respectively, red vertical lines as presented in Section 4.4) are not directly comparable. However, the full range of possible cutoffs for both values are plotted. Each panel shows the change in over-expressed GO terms with cutoff for a different value of the p parameter (prior estimate of expected proportion of interactors) used in the JHM analysis.

7 Correlation between methods

The Addinall et al. (2011) approach has its highest correlation with the IHM, followed by the JHM and then the REM. The REM correlates least well with the JHM while showing the same correlation with both the Addinall et al. (2011) approach and the IHM. The correlation between the IHM and the JHM is the largest observed between any of the methods, demonstrating the similarity of our Bayesian hierarchical methods.

Method	*Method*			
	Addinall et al. (2011) QFA	*REM QFA*	*IHM QFA*	*JHM QFA ($MDR \times MDP$)*
Addinall et al. (2011) QFA,	1	0.77	0.89	0.88
REM QFA,		1	0.77	0.75
IHM QFA,			1	0.95
JHM QFA ($MDR \times MDP$),				1

Table 7.1: Spearman's rank correlation coefficients for magnitudes from genetic independence, between Addinall et al. (2011), REM, IHM and JHM QFA methods

The $MDR \times MDP$ correlation plot of the JHM versus the Addinall et al. (2011) approach demonstrates the similarity (Pearson correlation=0.90) and differences between the two approaches in terms of $MDR \times MDP$. We can see how the results differ between the JHM and Addinall et al. (2011), with a kink at the origin due to the JHM allowing shrinkage of non-interacting genes towards the fitted line.

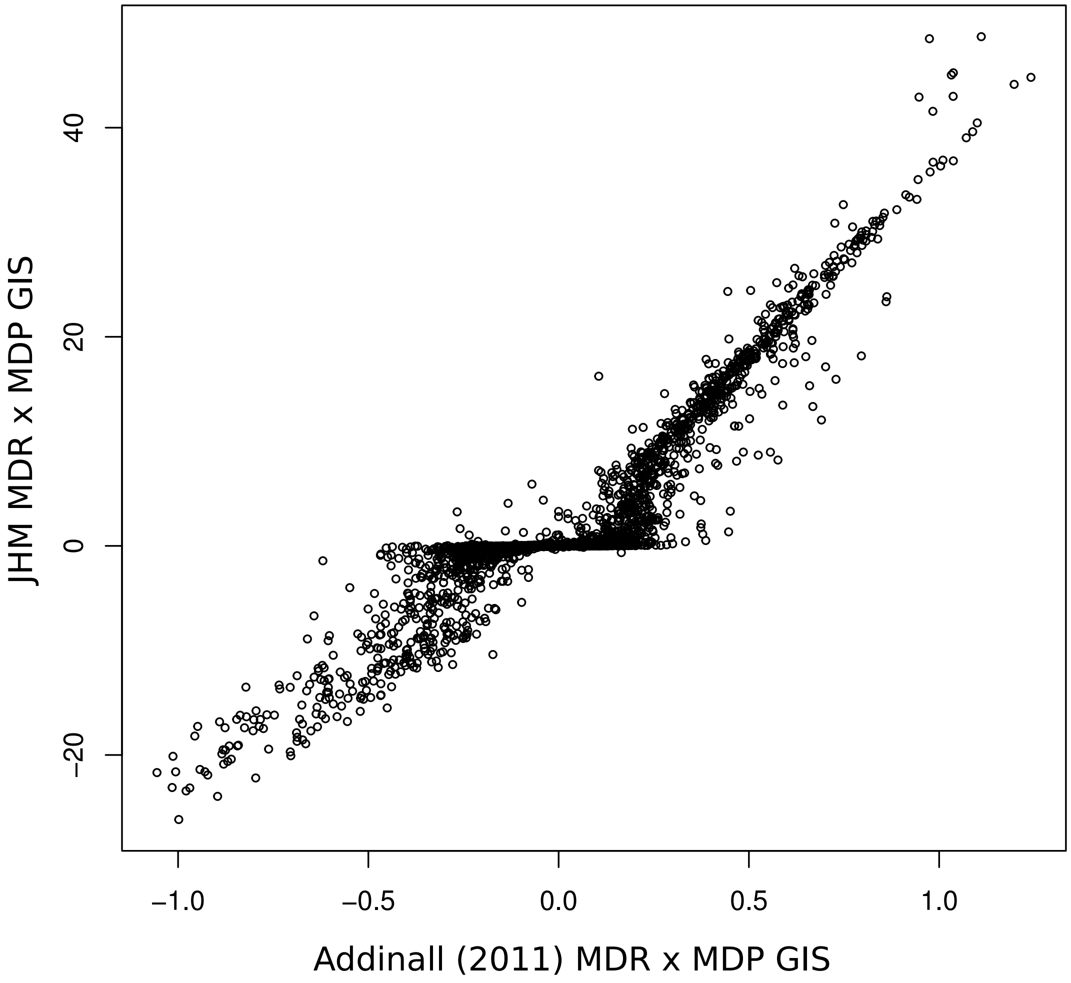

Figure 7.1: $MDR \times MDP$ genetic interaction correlation plot of JHM versus Addinall et al. (2011) (Pearson correlation=0.90).

8 A simulation study comparing specificity and sensitivity of the Addinall et al. (2011) approach, the SHM and the JHM

Since our understanding of biological processes is currently incomplete it is difficult to assess what proportion of genetic interactions identified by fitting any model to real biological data are real. If we don't know which interactions are true, we cannot know which of those interactions identified by any inference scheme are false positives. In order to compare the ability of each of our models to identify subtle, true interactions (sensitivity) while avoiding false positives (specificity), a separate simulation study was carried out (Section 4.3.6 http://arxiv.org/abs/1405.7091). Synthetic control and query datasets of similar size, quality and resolution to real QFA datasets, with known suppressors and enhancers of a simulated query mutation were constructed using a hierarchical simulation model consistent with the JHM. We used the JHM to simulate the synthetic dataset since it is the most detailed model we have available and the one which most closely matches the structure of QFA experiments. The Addinall et al. (2011) approach, the REM, the SHM and the JHM were each fit to the synthetic dataset and the lists of suppressors and enhancers as well as the list of all interactors generated by each method were compared with the list of known true interactors.

Sensitivity and specificity achieved with each of the models were presented in table 4.4 http://arxiv.org/abs/1405.7091. In summary, the simulation study showed that the JHM correctly identified a higher proportion of true interactions (314/430) than the Addinall et al. (2011) approach (220/430), while also identifying fewer false positives (JHM: 8, Addinall et al. (2011): 303).
